# Supplementary material for: Phylogeny and species delimitation of the genus Longgenacris and Fruhstorferiola viridifemorata species group (Orthoptera: Acrididae: Melanoplinae) based on molecular evidence
Source: PLoS One. 2020 Aug 26;15(8):e0237882. doi: 10.1371/journal.pone.0237882 (PMC7449498; doi:10.1371/journal.pone.0237882)
Supplement: S1 Table — (DOCX) [file pone.0237882.s001.docx]

**S1 Table. Materials involved in this study**

| Taxon | Voucher number | Locality, time and collector |
| --- | --- | --- |
| **Cealifera: Acridoidea: Acrididae; Melanoplinae** |  |  |
| *Fruhstorferiola viridifemorata* | gh001-008 | Qianmutian, Longwangshan, Anji County, Zhejiang Province, China; 14 September 2012; Rongsheng Lu. |
| *Fruhsforferiola omei* | gh085-087 | Wanniansi, Emeishan, Sichuan Province, China; 26 July 2011; Ruigang Yang. |
| *Fruhstorferiola huayinensis* | gl0095-0100 | Haopingshi, Taibaishan, Shaanxi, China; 13 July 2005; Zhaoqiang Qian |
|  | gl0227-0231 | Nanwutai, Chang’an, Shaanxi, China; 7 September 2008; Jianhua Huang |
|  | gl0232-0234 | Huayangchuan, Huayin, Shaanxi, China; 3 September 2008; Jianhua Huang |
|  | gl0235-0240 | Baiyunshan, Songxian, Henan, China; 17 August 2008; Jianhua Huang |
| *Fruhstorferiola kulinga* | gl0101-0106 | Hengshan, Hunan, China; 29 August 2007; Jianhua Huang |
|  | gl0107-0108 | Jingshan, Jingzhou, Hubei, China; 10 October 2007; Fuming Shi |
|  | gl0109-0112 | Longmenhe, Xingshan, Hubei, China; 4 August 2003; Yulin Zhong |
|  | gl0113-0115 | Gaozhai, Xing’an, Guangxi, China; 9 August 2006; Jianhua Huang |
| *Fruhstorferiola tonkinensis* | gh009-014 | Sanka, Minqiang, Longzhou County, Guangxi, China; 3 August 2012; Tao Wei. |
|  | gh040-044 | Gaoji, Sanjiang County, Guangxi, China; 11 July 2009; Jianhua Huang. |
|  | gh055-059 | Longjiang, Nonggang, Longzhou County, Guangxi, China; 27 August 2012; Tao Wei. |
|  | gh154-158 | Nonggang, Longzhou County, Guangxi, China; 26 July 2012; Tao Wei. |
|  | gl0089-0094 | Yong'an, Xing'an, Guangxi, China; 2 July 2006; Jianhua Huang |
| *Longgenacris rufiantennus* | gh080-084,  113-117, 123-127 | Xiaolong, Yizhou, Hechi, Guangxi, China; 7 July 2012; Tao Wei. |
| *Longgenacris maculacarina* | gh015-019, | Longjiang, Nonggang, Longzhou County, Guangxi, China; 27 July 2012; Tao Wei. |
|  | gh144-148 | Longfang, Nonggang, Longzhou County, Guangxi, China; 25 August 2012; Tao Wei. |
|  | gh159-163 | Longshi, Nonggang, Longzhou County, Guangxi, China; 28 July 2012; Tao Wei. |
| *Paratonkinacris vittifemoralis* | gh045-049  gl0247-0251 | Gaozhai, Xing'an County, Guangxi, China; 8 July 2009; Jianhua Huang. |
| *Emeiacris maculata* | gh075-079, 088-092 | Hongchunping, Emeishan, Sichuan County, China; 27 July 2011; Ruigang Yang. |
|  | gl0241-0246 | Hengshan, Hunan, China; 29 August 2008; Jianhua Huang. |
| *Tonkinacris sinensis* | gl0257-0261 | Yong'an, Xing'an County, Guangxi, China; 2 July 2006; Jianhua Huang. |
| *Ognevia longipennis* | gl0252-0256 | Yangjiaping, Zhuolu, Hebei, China; 20 August 2005; Yuan Huang |
| **Coptacrinae** |  |  |
| *Apalacris tonkinensis* | gh164-168 | Longjiang, Nonggang, Longzhou County, Guangxi, China; 27 August 2012; Tao Wei. |
|  | gh207-211 | Longshi, Nonggang, Longzhou County, Guangxi, China; 29 July 2012; Tao Wei. |
| **Tetrigoidea: Tetrigidae** |  |  |
| *Ergatettix dorsiferus* | gh247 | Fangchenggang, Guangxi, China; 18 December 2013; Chunwen Lu. |
| **Ensifera: Tettigonioidea: Tettigoniidae** |  |  |
| *Conocephalus longipennis* | gh242-243 | Lengjiacun, Guilin, Guangxi, China; 23 October 2013; Tao Wei. |
