# Supplementary material for: Phylogeny and species delimitation of the genus Longgenacris and Fruhstorferiola viridifemorata species group (Orthoptera: Acrididae: Melanoplinae) based on molecular evidence
Source: PLoS One. 2020 Aug 26;15(8):e0237882. doi: 10.1371/journal.pone.0237882 (PMC7449498; doi:10.1371/journal.pone.0237882)
Supplement: S2 Table — (DOCX) [file pone.0237882.s002.docx]

**S2 Table. Mapping table between GenBank accession numbers and voucher numbers**

| Voucher number | GenBank accession number | | | Voucher number | GenBank accession number | | |
| --- | --- | --- | --- | --- | --- | --- | --- |
|  | COI | ITS1 | ITS2 |  | COI | ITS1 | ITS2 |
| gh001 | MH934098 | MH934130 | MH934130 | gh083 | MH934109 | MH934136 | MH934136 |
| gh002 | MH934099 | MH934130 | MH934130 | gh084 | MH934109 | MH934140 | MH934140 |
| gh003 | MH934098 | MH934130 | MH934130 | gh113 | MH934109 | MH934140 | MH934140 |
| gh004 | MH934100 | MH934130 | MH934130 | gh114 | MH934109 | MH934140 | MH934140 |
| gh005 | MH934098 | MH934131 | MH934131 | gh115 | MH934116 | MH934140 | MH934140 |
| gh006 | MH934098 | MH934132 | MH934132 | gh116 | MH934109 | MH934140 | MH934140 |
| gh007 | MH934101 | MH934133 | MH934133 | gh117 | MH934116 | MH934140 | MH934140 |
| gh008 | MH934102 | MH934134 | MH934134 | gh123 | MH934109 | MH934136 | MH934136 |
| gh085 | MH934103 | MH934133 | MH934133 | gh124 | MH934115 | MH934140 | MH934140 |
| gh086 | MH934104 | MH934135 | MH934135 | gh125 | MH934109 | MH934140 | MH934140 |
| gh087 | MH934105 | MH934133 | MH934133 | gh126 | MH934109 | MH934140 | MH934140 |
| gl0095 | KC139861* | MH934142 | MH934142 | gh127 | MH934109 | MH934140 | MH934140 |
| gl0096 | KC139862* | MH934142 | MH934142 | gh015 | MH934117 | MH934156 | MH934156 |
| gl0097 | KC139863* | MH934143 | MH934143 | gh016 | MH934117 | MH934157 | MH934157 |
| gl0098 | KC139864* | MH934142 | MH934142 | gh017 | MH934117 | MH934158 | MH934158 |
| gl0099 | KC139865* | MH934142 | MH934142 | gh018 | MH934117 | MH934158 | MH934158 |
| gl0100 | KC139866* | MH934142 | MH934142 | gh019 | MH934117 | MH934159 | MH934159 |
| gl0227 | KC139861* | MH934144 | MH934144 | gh144 | MH934118 | MH934160 | MH934160 |
| gl0228 | KC139867* | MH934144 | MH934144 | gh145 | MH934117 | MH934160 | MH934160 |
| gl0229 | KC139868* | MH934144 | MH934144 | gh146 | MH934118 | MH934160 | MH934160 |
| gl0230 | KC139869* | MH934144 | MH934144 | gh147 | MH934117 | MH934160 | MH934160 |
| gl0231 | KC139861* | MH934144 | MH934144 | gh148 | MH934117 | MH934160 | MH934160 |
| gl0232 | KC139865* | MH934142 | MH934142 | gh159 | MH934117 | MH934160 | MH934160 |
| gl0233 | KC139861* | MH934142 | MH934142 | gh160 | MH934118 | MH934160 | MH934160 |
| gl0234 | KC139865* | MH934142 | MH934142 | gh161 | MH934119 | MH934160 | MH934160 |
| gl0235 | KC139870* | MH934142 | MH934142 | gh162 | MH934117 | MH934160 | MH934160 |
| gl0236 | KC139871* | MH934142 | MH934142 | gh163 | MH934120 | MH934160 | MH934160 |
| gl0237 | KC139872* | MH934142 | MH934142 | gh045 | MH934121 | MH934161 | MH934161 |
| gl0238 | KC139863* | MH934142 | MH934142 | gh046 | MH934121 | MH934161 | MH934161 |
| gl0239 | KC139872* | MH934142 | MH934142 | gh047 | MH934121 | MH934161 | MH934161 |
| gl0240 | KC139873* | MH934142 | MH934142 | gh048 | MH934121 | MH934161 | MH934161 |
| gl0101 | KC139874* | MH934145 | MH934145 | gh049 | MH934122 | MH934161 | MH934161 |
| gl0102 | KC139875* | MH934146 | MH934146 | gl0247 | KC139857* | MH934162 | MH934162 |
| gl0103 | KC139876* | MH934147 | MH934147 | gl0248 | KC139858* | MH934161 | MH934161 |
| gl0104 | KC139877* | MH934148 | MH934148 | gl0249 | KC139858* | MH934161 | MH934161 |
| gl0105 | KC139875* | MH934149 | MH934149 | gl0250 | KC139859* | MH934161 | MH934161 |
| gl0106 | KC139875* | MH934147 | MH934147 | gl0251 | KC139860* | MH934163 | MH934163 |
| gl0107 | KC139878* | MH934150 | MH934150 | gh075 | MH934123 | MH934164 | MH934164 |
| gl0108 | KC139879* | MH934147 | MH934147 | gh076 | MH934123 | MH934165 | MH934165 |
| gl0109 | KC139880* | MH934147 | MH934147 | gh077 | MH934123 | MH934166 | MH934166 |
| gl0110 | KC139881* | MH934151 | MH934151 | gh078 | MH934123 | MH934167 | MH934167 |
| gl0111 | KC139882* | MH934151 | MH934151 | gh079 | MH934123 | MH934168 | MH934168 |
| gl0112 | KC139883* | MH934142 | MH934142 | gh088 | MH934123 | MH934169 | MH934169 |
| gl0113 | KC139884* | MH934152 | MH934152 | gh089 | MH934123 | MH934170 | MH934170 |
| gl0114 | KC139885* | MH934153 | MH934153 | gh090 | MH934123 | MH934161 | MH934161 |
| gl0115 | KC139875* | MH934154 | MH934154 | gh091 | MH934124 | MH934171 | MH934171 |
| gh009 | MH934106 | MH934136 | MH934136 | gh092 | MH934123 | MH934170 | MH934170 |
| gh010 | MH934106 | MH934136 | MH934136 | gl0241 | KC139851* | MH934172 | MH934172 |
| gh011 | MH934106 | MH934137 | MH934137 | gl0242 | KC139852* | MH934172 | MH934172 |
| gh012 | MH934107 | MH934136 | MH934136 | gl0243 | KC139853* | MH934172 | MH934172 |
| gh013 | MH934108 | MH934136 | MH934136 | gl0244 | KC139854* | MH934172 | MH934172 |
| gh014 | MH934106 | MH934138 | MH934138 | gl0245 | KC139855* | MH934173 | MH934173 |
| gh040 | MH934109 | MH934136 | MH934136 | gl0246 | KC139856* | MH934173 | MH934173 |
| gh041 | MH934109 | MH934139 | MH934139 | gl0257 | KC139972* | MH934174 | MH934174 |
| gh042 | MH934110 | MH934136 | MH934136 | gl0258 | KC139973* | MH934175 | MH934175 |
| gh043 | MH934111 | MH934136 | MH934136 | gl0259 | KC139973* | MH934174 | MH934174 |
| gh044 | MH934112 | MH934136 | MH934136 | gl0260 | KC139972* | MH934175 | MH934175 |
| gh055 | MH934113 | MH934136 | MH934136 | gl0261 | KC139973* | MH934174 | MH934174 |
| gh056 | MH934107 | MH934136 | MH934136 | gl0252 | KC139896* | MH934176 | MH934176 |
| gh057 | MH934113 | MH934136 | MH934136 | gl0253 | KC139897* | MH934177 | MH934177 |
| gh058 | MH934114 | MH934136 | MH934136 | gl0254 | KC139896* | MH934177 | MH934177 |
| gh059 | MH934113 | MH934136 | MH934136 | gl0255 | KC139896* | MH934178 | MH934178 |
| gh154 | MH934113 | MH934140 | MH934140 | gl0256 | KC139898* | MH934178 | MH934178 |
| gh155 | MH934113 | MH934140 | MH934140 | gh164 | MH934125 | MH934179 | MH934179 |
| gh156 | MH934106 | MH934140 | MH934140 | gh165 | MH934125 | MH934180 | MH934180 |
| gh157 | MH934106 | MH934140 | MH934140 | gh166 | MH934126 | MH934180 | MH934180 |
| gh158 | MH934106 | MH934140 | MH934140 | gh167 | MH934125 | MH934181 | MH934181 |
| gl0089 | KC139886* | MH934141 | MH934141 | gh168 | MH934125 | MH934180 | MH934180 |
| gl0090 | KC139887* | MH934140 | MH934140 | gh207 | MH934125 | MH934182 | MH934182 |
| gl0091 | KC139888* | MH934140 | MH934140 | gh208 | MH934126 | MH934183 | MH934183 |
| gl0092 | KC139889* | MH934140 | MH934140 | gh209 | MH934126 | MH934181 | MH934181 |
| gl0093 | KC139890* | MH934140 | MH934140 | gh210 | MH934125 | MH934181 | MH934181 |
| gl0094 | KC139890* | MH934140 | MH934140 | gh211 | MH934125 | MH934184 | MH934184 |
| gh080 | MH934109 | MH934155 | MH934155 | gh247 | MH934127 | MH934185 | MH934185 |
| gh081 | MH934115 | MH934140 | MH934140 | gh242 | MH934128 | MH934186 | MH934186 |
| gh082 | MH934109 | MH934136 | MH934136 | gh243 | MH934129 | MH934186 | MH934186 |

Note. The asterisk symbol "*" indicates the sequence generated in the previous study published by Huang *et al* (2013).
