# Supplementary material for: Phylogeny and species delimitation of the genus Longgenacris and Fruhstorferiola viridifemorata species group (Orthoptera: Acrididae: Melanoplinae) based on molecular evidence
Source: PLoS One. 2020 Aug 26;15(8):e0237882. doi: 10.1371/journal.pone.0237882 (PMC7449498; doi:10.1371/journal.pone.0237882)
Supplement: S3 Table — (DOCX) [file pone.0237882.s003.docx]

**S3 Table. Intraspecific variations calculated from different datasets.**

| Species/populations | Intraspecific variations (mean distance) | | |
| --- | --- | --- | --- |
|  | COI | ITS1 | ITS2 |
| *Fruhstorferiola viridifemorata* | 0-1.23% (0.60%) | 0-0.64% (0.19%) | 0-1.12% (0.49%) |
| *Fruhstorferiola omei* | 0.15-0.77% (0.51%) | 0 (0) | 0-0.84% (0.56%) |
| *Fruhstorferiola huayinensis* | 0-1.85% (0.57%) | 0 (0) | 0-0.84%　(0.19%) |
| *F. huayinensis -Taibaishan* | 0.15-0.77% (0.53%) | 0 (0) | 0-0.56% (0.19%) |
| *F. huayinensis -Nanwutai* | 0-0.61% (0.34%) | 0 (0) | 0 (0) |
| *F. huayinensis -Huayangchuan* | 0-0.15% (0.10%) | 0 (0) | 0 (0) |
| *F. huayinensis -Baiyunshan* | 0-1.54% (0.77%) | 0 (0) | 0-0.28% (0.09%) |
| *Fruhstorferiola kulinga* | 0-**2.97%** (1.02%) | 0-**1.29%** (0.33%) | 0-0.56% (0.11%) |
| *F. kulinga -Hengshan* | 0-0.30% (0.15) | 0-0.32% (0.16%) | 0-0.28% (0.15%) |
| *F. kulinga -Jingshan* | 0.46% (0.46%) | 0.48% (0.48%) | 0 (0) |
| *F. kulinga -Longmenhe* | 0.46-**2.33%** (1.60%) | 0-0.32% (0.16%) | 0 (0) |
| *F. kulinga -Gaozhai* | 0.15-0.46% (0.31%) | 0.32-0.64% (0.53%) | 0-0.28% (0.19%) |
| *Fruhstorferiola tonkinensis* | 0-1.08% (0.45%) | 0-0.82% (0.09%) | 0-0.84% (0.30%) |
| *Longgenacris rufiantennus* | 0-0.46% (0.11%) | 0 (0) | 0-1.41% (0.35%) |
| *Longgenacris maculacarina* | 0-0.61% (0.17%) | 0-3.08% (0.41%) | 0-1.70% (0.39%) |
| *Paratonkinacris vittifemoralis* | 0-1.38% (0.46%) | 0 (0) | 0-0.84% (0.21%) |
| *Emeiacris maculata* | 0-**4.73%** (2.28%) | 0-0.82% (0.33%) | 0-1.41% (0.40%) |
| *E. maculata -Emeishan* | 0-0.15% (0.03%) | 0-0.66% (0.26%) | 0-1.41%(0.49%) |
| *E. maculata -Hengshan* | 0.15-0.61% (0.36%) | 0 (0) | 0-0.56% (0.30%) |
| *Tonkinacris sinensis* | 0-0.30% (0.18%) | 0 (0) | 0-0.28% (0.17%) |
| *Ognevia longipennis* | 0-0.30% (0.12%) | 0-0.34% (0.20%) | 0-0.28% (0.11%) |
| *Apalacris tonkinensis* | 0-0.15% (0.07%) | 0 (0) | 0-0.60% (0.32%) |
