# Supplementary material for: Phylogeny and species delimitation of the genus Longgenacris and Fruhstorferiola viridifemorata species group (Orthoptera: Acrididae: Melanoplinae) based on molecular evidence
Source: PLoS One. 2020 Aug 26;15(8):e0237882. doi: 10.1371/journal.pone.0237882 (PMC7449498; doi:10.1371/journal.pone.0237882)
Supplement: S4 Table — (DOCX) [file pone.0237882.s004.docx]

**S4 Table. Mean genetic distances between species calculated from COI alignment**

|  | *F. vir* | *F. omei* | *F. hua* | *F. kul* | *F. ton* | *L. ruf* | *L. mac* | *P. vit* | *Em. mac* | *T. sin* | *O. lon* | *A. ton* | *Er. dor* |
| --- | --- | --- | --- | --- | --- | --- | --- | --- | --- | --- | --- | --- | --- |
| *F. omei* | 0.0100 |  |  |  |  |  |  |  |  |  |  |  |  |
| *F. hua* | 0.0191 | 0.0203 |  |  |  |  |  |  |  |  |  |  |  |
| *F. kul* | 0.0148 | 0.0165 | 0.0112 |  |  |  |  |  |  |  |  |  |  |
| *F. ton* | 0.0553 | 0.0608 | 0.0568 | 0.0605 |  |  |  |  |  |  |  |  |  |
| *L. ruf* | 0.0544 | 0.0600 | 0.0560 | 0.0596 | 0.0033 |  |  |  |  |  |  |  |  |
| *L. mac* | 0.0680 | 0.0695 | 0.0655 | 0.0711 | 0.0740 | 0.0733 |  |  |  |  |  |  |  |
| *P. vit* | 0.0673 | 0.0680 | 0.0713 | 0.0698 | 0.0743 | 0.0734 | 0.0731 |  |  |  |  |  |  |
| *Em. mac* | 0.0729 | 0.0753 | 0.0748 | 0.0750 | 0.0843 | 0.0834 | 0.0781 | 0.0516 |  |  |  |  |  |
| *T. sin* | 0.0780 | 0.0773 | 0.0745 | 0.0735 | 0.0949 | 0.0942 | 0.0765 | 0.0780 | 0.0788 |  |  |  |  |
| *O. lon* | 0.1081 | 0.1141 | 0.1151 | 0.1125 | 0.1089 | 0.1087 | 0.1233 | 0.1172 | 0.1127 | 0.1224 |  |  |  |
| *A. ton* | 0.1835 | 0.1887 | 0.1788 | 0.1835 | 0.1905 | 0.1900 | 0.1842 | 0.1773 | 0.1696 | 0.1928 | 0.1891 |  |  |
| *Er. dor* | 0.3072 | 0.3074 | 0.3167 | 0.3123 | 0.3188 | 0.3181 | 0.3098 | 0.3059 | 0.3171 | 0.2911 | 0.2930 | 0.3002 |  |
| *C. lon* | 0.2854 | 0.2858 | 0.2806 | 0.2833 | 0.2782 | 0.2765 | 0.2823 | 0.2772 | 0.2825 | 0.2664 | 0.2852 | 0.2741 | 0.3239 |

Note. F. vir: *Fruhstorferiola viridifemorata*; F. omei: *Fruhstorferiola omei*; F. hua: *Fruhstorferiola* *huayinensis*; F. kul : *Fruhstorferiola kulinga;* F. ton: *Fruhstorferiola tonkinensis*; L. ruf: *Longgenacris rufiantennus*; L. mac: *Longgenacris maculacarina*; P. vit: *Paratonkinacris vittifemoralis*; Em. mac: *Emeiacris maculata*; T. sin: *Tonkinacris sinensis*; O. lon: *Ognevia longipennis*; A. ton: Apalacris tonkinensis; Er. dor: *Ergatettix dorsiferus*; C. lon: *Conocephalus longipennis*.
