# Supplementary material for: Phylogeny and species delimitation of the genus Longgenacris and Fruhstorferiola viridifemorata species group (Orthoptera: Acrididae: Melanoplinae) based on molecular evidence
Source: PLoS One. 2020 Aug 26;15(8):e0237882. doi: 10.1371/journal.pone.0237882 (PMC7449498; doi:10.1371/journal.pone.0237882)
Supplement: S5 Table — (DOCX) [file pone.0237882.s005.docx]

**S5 Table. Mean genetic distances between species calculated from ITS1 alignment**

|  | *F. vir* | *F. omei* | *F. hua* | *F. kul* | *F. ton* | *L. ruf* | *L. mac* | *P. vit* | *Em. mac* | *T. sin* | *O. lon* | *A. ton* | *Er. dor* |
| --- | --- | --- | --- | --- | --- | --- | --- | --- | --- | --- | --- | --- | --- |
| *F. omei* | 0.0018 |  |  |  |  |  |  |  |  |  |  |  |  |
| *F. hua* | 0.0010 | 0.0016 |  |  |  |  |  |  |  |  |  |  |  |
| *F. kul* | 0.0042 | 0.0045 | 0.0033 |  |  |  |  |  |  |  |  |  |  |
| *F. ton* | 0.0064 | 0.0070 | 0.0053 | 0.0079 |  |  |  |  |  |  |  |  |  |
| *L. ruf* | 0.0060 | 0.0066 | 0.0050 | 0.0075 | 0.0005 |  |  |  |  |  |  |  |  |
| *L. mac* | 0.0267 | 0.0248 | 0.0265 | 0.0303 | 0.0323 | 0.0319 |  |  |  |  |  |  |  |
| *P. vit* | 0.0270 | 0.0251 | 0.0268 | 0.0307 | 0.0314 | 0.0310 | 0.0234 |  |  |  |  |  |  |
| *Em. mac* | 0.0293 | 0.0273 | 0.0290 | 0.0323 | 0.0318 | 0.0314 | 0.0268 | 0.0025 |  |  |  |  |  |
| *T. sin* | 0.0231 | 0.0212 | 0.0229 | 0.0266 | 0.0257 | 0.0253 | 0.0251 | 0.0167 | 0.0190 |  |  |  |  |
| *O. lon* | 0.0360 | 0.0340 | 0.0358 | 0.0379 | 0.0393 | 0.0389 | 0.0405 | 0.0258 | 0.0269 | 0.0214 |  |  |  |
| *A. ton* | 0.1345 | 0.1327 | 0.1348 | 0.1370 | 0.1391 | 0.1386 | 0.1439 | 0.1278 | 0.1285 | 0.1290 | 0.1389 |  |  |
| *Er. dor* | 0.3829 | 0.3802 | 0.3833 | 0.3827 | 0.3869 | 0.3864 | 0.3736 | 0.3607 | 0.3639 | 0.3720 | 0.4055 | 0.4057 |  |
| *C. lon* | 0.5096 | 0.5064 | 0.5102 | 0.5148 | 0.5231 | 0.5224 | 0.5234 | 0.4974 | 0.4995 | 0.5055 | 0.5270 | 0.5338 | 0.5688 |

Note. F. vir: *Fruhstorferiola viridifemorata*; F. omei: *Fruhstorferiola omei*; F. hua: *Fruhstorferiola* *huayinensis*; F. kul : *Fruhstorferiola kulinga;* F. ton: *Fruhstorferiola tonkinensis*; L. ruf: *Longgenacris rufiantennus*; L. mac: *Longgenacris maculacarina*; P. vit: *Paratonkinacris vittifemoralis*; Em. mac: *Emeiacris maculata*; T. sin: *Tonkinacris sinensis*; O. lon: *Ognevia longipennis*; A. ton: Apalacris tonkinensis; Er. dor: *Ergatettix dorsiferus*; C. lon: *Conocephalus longipennis*.
