# Supplementary material for: Phylogeny and species delimitation of the genus Longgenacris and Fruhstorferiola viridifemorata species group (Orthoptera: Acrididae: Melanoplinae) based on molecular evidence
Source: PLoS One. 2020 Aug 26;15(8):e0237882. doi: 10.1371/journal.pone.0237882 (PMC7449498; doi:10.1371/journal.pone.0237882)
Supplement: S6 Table — (DOCX) [file pone.0237882.s006.docx]

**S6 Table. Mean genetic distances between species calculated from ITS2 alignment**

|  | *F. vir* | *F. omei* | *F. hua* | *F. kul* | *F. ton* | *L. ruf* | *L. mac* | *P. vit* | *Em. mac* | *T. sin* | *O. lon* | *A. ton* | *Er. dor* |
| --- | --- | --- | --- | --- | --- | --- | --- | --- | --- | --- | --- | --- | --- |
| *F. omei* | 0.0076 |  |  |  |  |  |  |  |  |  |  |  |  |
| *F. hua* | 0.0071 | 0.0037 |  |  |  |  |  |  |  |  |  |  |  |
| *F. kul* | 0.0063 | 0.0032 | 0.0017 |  |  |  |  |  |  |  |  |  |  |
| *F. ton* | 0.0135 | 0.0096 | 0.0122 | 0.0120 |  |  |  |  |  |  |  |  |  |
| *L. ruf* | 0.0142 | 0.0087 | 0.0107 | 0.0106 | 0.0037 |  |  |  |  |  |  |  |  |
| *L. mac* | 0.0252 | 0.0195 | 0.0216 | 0.0215 | 0.0196 | 0.0183 |  |  |  |  |  |  |  |
| *P. vit* | 0.0164 | 0.0158 | 0.0172 | 0.0166 | 0.0167 | 0.0176 | 0.0178 |  |  |  |  |  |  |
| *Em. mac* | 0.0164 | 0.0147 | 0.0158 | 0.0153 | 0.0167 | 0.0168 | 0.0170 | 0.0032 |  |  |  |  |  |
| *T. sin* | 0.0191 | 0.0120 | 0.0135 | 0.0135 | 0.0134 | 0.0111 | 0.0110 | 0.0124 | 0.0110 |  |  |  |  |
| *O. lon* | 0.0193 | 0.0175 | 0.0210 | 0.0206 | 0.0140 | 0.0158 | 0.0214 | 0.0158 | 0.0166 | 0.0164 |  |  |  |
| *A. ton* | 0.0958 | 0.0922 | 0.0930 | 0.0924 | 0.0960 | 0.0959 | 0.1090 | 0.1038 | 0.1030 | 0.0968 | 0.1044 |  |  |
| *Er. dor* | 0.4951 | 0.4852 | 0.4881 | 0.4874 | 0.4822 | 0.4784 | 0.4850 | 0.4976 | 0.4949 | 0.4795 | 0.4901 | 0.5053 |  |
| *C. lon* | 0.6178 | 0.6190 | 0.6204 | 0.6199 | 0.6136 | 0.6140 | 0.6265 | 0.6252 | 0.6241 | 0.6197 | 0.6352 | 0.5831 | 0.7930 |

Note. F. vir: *Fruhstorferiola viridifemorata*; F. omei: *Fruhstorferiola omei*; F. hua: *Fruhstorferiola* *huayinensis*; F. kul : *Fruhstorferiola kulinga;* F. ton: *Fruhstorferiola tonkinensis*; L. ruf: *Longgenacris rufiantennus*; L. mac: *Longgenacris maculacarina*; P. vit: *Paratonkinacris vittifemoralis*; Em. mac: *Emeiacris maculata*; T. sin: *Tonkinacris sinensis*; O. lon: *Ognevia longipennis*; A. ton: Apalacris tonkinensis; Er. dor: *Ergatettix dorsiferus*; C. lon: *Conocephalus longipennis*.
