# Supplementary material for: Phylogeny and species delimitation of the genus Longgenacris and Fruhstorferiola viridifemorata species group (Orthoptera: Acrididae: Melanoplinae) based on molecular evidence
Source: PLoS One. 2020 Aug 26;15(8):e0237882. doi: 10.1371/journal.pone.0237882 (PMC7449498; doi:10.1371/journal.pone.0237882)
Supplement: S7 Table — (DOCX) [file pone.0237882.s007.docx]

**S7 Table. Haplotyptes of COI detected from samples of *F. viridifemorata* and *F. tontinensis*+*L. rufiantennus* groups**

| Haplotype number | Individuals involved | Haplotype number | Individuals involved |
| --- | --- | --- | --- |
| 1 | ***F. viridifemorata***  **Longwangshan, Zhejiang:** gh001, gh003, gh005, gh006 | 16 | ***F. huayinensis***  **Nanwutai, Shaanxi:** gl0229. |
| 2 | ***F. viridifemorata***  **Longwangshan, Zhejiang:** gh002. | 17 | ***F. huayinensis***  **Nanwutai, Shaanxi:** gl0230. |
| 3 | ***F. viridifemorata***  **Longwangshan, Zhejiang:** gh004. | 18 | ***F. huayinensis***  **Baiyunshan, Henan:** gl0235. |
| 4 | ***F. viridifemorata***  **Longwangshan, Zhejiang:** gh007. | 19 | ***F. huayinensis***  **Baiyunshan, Henan:** gl0236. |
| 5 | ***F. viridifemorata***  **Longwangshan, Zhejiang:** gh008. | 20 | ***F. huayinensis***  **Baiyunshan, Henan:** gl0237, gl0239. |
| 6 | ***F. omei***  **Emeishan, Sichuan:** gh085. | 21 | ***F. huayinensis***  **Baiyunshan, Henan:** gl0240. |
| 7 | ***F. omei***  **Emeishan, Sichuan:** gh086. | 22 | ***F. kulinga***  **Hengshan, Hunan:** gl0101 |
| 8 | ***F. omei***  **Emeishan, Sichuan:** gh087. | 23 | ***F. kulinga***  **Hengshan, Hunan:** gl0102, gl0105, gl0106. **Gaozhai, Guangxi:** gl0115. |
| 9 | ***F. huayinensis***  **Taibaishan, Shaanxi:** gl0095. **Nanwutai, Shaanxi:** gl0227, gl0231. **Huayangchuan, Shaanxi:** gl0233. | 24 | ***F. kulinga***  **Hengshan, Hunan:** gl0103. |
| 10 | ***F. huayinensis***  **Taibaishan, Shaanxi:** gl0096. | 25 | ***F. kulinga***  **Hengshan, Hunan:** gl0104. |
| 11 | ***F. huayinensis***  **Taibaishan, Shaanxi:** gl0097. **Baiyunshan, Henan:** gl0238. | 26 | ***F. kulinga***  **Jingshan, Hubei:** gl0107. |
| 12 | ***F. huayinensis***  **Taibaishan, Shaanxi:** gl0098. | 27 | ***F. kulinga***  **Jingshan, Hubei:** gl0108. |
| 13 | ***F. huayinensis***  **Taibaishan, Shaanxi:** gl0099. **Huayangchuan, Shaanxi:** gl0232, gl0234. | 28 | ***F. kulinga***  **Xingshan, Hubei:** gl0109. |
| 14 | ***F. huayinensis***  **Taibaishan, Shaanxi:** gl0100. | 29 | ***F. kulinga***  **Xingshan, Hubei:** gl0110. |
| 15 | ***F. huayinensis***  **Nanwutai, Shaanxi:** gl0228. | 30 | ***F. kulinga***  **Xingshan, Hubei:** gl0111. |

**S7 Table. (continued)**

| Haplotype number | Individuals involved | Haplotype number | Individuals involved |
| --- | --- | --- | --- |
| 31 | ***F. kulinga***  **Xingshan, Hubei:** gl0112. | 40 | ***F. tontinensis***  **Gaoji, Sanjiang:** gh044.  **Yong'an, Guangxi:** gl0093, gl0094. |
| 32 | ***F. kulinga:***  **Gaozhai, Guangxi:** gl0113. | 41 | ***F. tontinensis***  **Longjiang, Longzhou:** gh055, gh057, gh059.  **Nonggang, Longzhou:** gh154, gh155. |
| 33 | ***F. kulinga:***  **Gaozhai, Guangxi:** gl0114. | 42 | ***F. tontinensis***  **Longjiang, Longzhou:** gh058. |
| 34 | ***F. tontinensis***  **Sanka, Longzhou:** gh009, gh010, gh011, gh014.  **Nonggang, Longzhou:** gh156, gh157, gh158. | 43 | ***F. tontinensis***  **Yong'an, Guangxi:** gl0090. |
| 35 | ***F. tontinensis***  **Sanka, Longzhou:** gh012; **Longjiang, Longzhou:** gh056. | 44 | ***F. tontinensis:***  **Yong'an, Guangxi:** gl0091. |
| 36 | ***F. tontinensis***  **Sanka, Longzhou:** gh013. | 45 | ***F. tontinensis:***  **Yong'an, Guangxi:** gl0092. |
| 37 | ***F. tontinensis***  **Gaoji, Sanjiang:** gh040, gh041; **Yong'an, Guangxi:** gl0089.  ***L. rufiantennus***  **Xiaolong, Guangxi:** gh080, gh082 , gh083 , gh084 , gh113 , gh114 , gh116 , gh123 , gh125 , gh126 , gh127. | 46 | ***L. rufiantennus***  **Xiaolong, Guangxi:** gh081, gh124. |
| 38 | ***F. tontinensis***  **Gaoji, Sanjiang:** gh042. | 47 | ***L. rufiantennus***  **Xiaolong, Guangxi:** gh115, gh117. |
| 39 | ***F. tontinensis***  **Gaoji, Sanjiang:** gh043. |  |  |
