# Supplementary material for: Phylogeny and species delimitation of the genus Longgenacris and Fruhstorferiola viridifemorata species group (Orthoptera: Acrididae: Melanoplinae) based on molecular evidence
Source: PLoS One. 2020 Aug 26;15(8):e0237882. doi: 10.1371/journal.pone.0237882 (PMC7449498; doi:10.1371/journal.pone.0237882)
Supplement: S9 Table — (DOCX) [file pone.0237882.s009.docx]

**Table S9. Haplotyptes of ITS2 detected from samples of *F. viridifemorata* and *F. tontinensis*+*L. rufiantennus* groups**

| Haplotype number | Individuals involved | Haplotype number | Individuals involved |
| --- | --- | --- | --- |
| 1 | ***Fruhstorferiola viridifemorata***  **Longwangshan, Zhejiang:** gh001, gh002, gh003, gh004, gh005. | 6 | ***Fruhstorferiola huayinensis***  **Taibaishan, Shaanxi:** gl0097. |
| 2 | ***Fruhstorferiola viridifemorata***  **Longwangshan, Zhejiang:** gh006. | 7 | ***Fruhstorferiola huayinensis***  **Nanwutai, Shaanxi:** gl0227, gl0228, gl0229, gl0230, gl0231. |
| 3 | ***Fruhstorferiola viridifemorata***  **Longwangshan, Zhejiang:** gh007.  ***Fruhstorferiola_omei***  **Emeishan, Sichuan:** gh085, gh087.  ***Fruhstorferiola huayinensis***  **Taibaishan, Shaanxi:** gl0095, gl0096, gl0098, gl0099, gl0100.  **Huayangchuan, Shaanxi:** gl0232, gl0233, gl0234.  **Baiyunshan, Henan:** gl0235, gl0236, gl0237, gl0238, gl0239,  ***Fruhstorferiola kulinga***  **Hengshan, Hunan:** gl0101, gl0103, gl0105, gl0106.  **Jingshan, Hubei:** gl0107, gl0108.  **Xingshan, Hubei:** gl0109, gl0110, gl0112.  **Gaozhai, Guangxi:** gl0113, gl0114, | 8 | ***Fruhstorferiola huayinensis***  **Baiyunshan, Henan:** gl0240. |
| 4 | ***Fruhstorferiola viridifemorata***  **Longwangshan, Zhejiang:** gh008. | 9 | ***Fruhstorferiola kulinga***  **Hengshan, Hunan:** gl0102, gl0104. |
| 5 | ***Fruhstorferiola omei***  **Emeishan, Sichuan:** gh086. | 10 | ***Fruhstorferiola kulinga***  **Gaozhai, Guangxi:** gl0115. |

**Table S9. (continued)**

| Haplotype number | Individuals involved | Haplotype number | Individuals involved |
| --- | --- | --- | --- |
| 11 | ***Fruhstorferiola tonkinensis***  **Sanka, Longzhou:** gh009, gh010, gh011, gh012, gh013, gh014.  **Gaoji, Sanjiang:** gh040, gh042, gh043, gh044.  **Longjiang, Longzhou:** gh055, gh056, gh057, gh058, gh059.  ***Longgenacris rufiantennus***  **Xiaolong, Yizhou:** gh082, gh083, gh123. | 13 | ***Fruhstorferiola tonkinensis***  **Nonggang, Longzhou:** gh154, gh155, gh156, gh157, gh158.  **Yong'an, Xing'an:** gl0089, gl0090, gl0091, gl0092, gl0093, gl0094.  ***Longgenacris rufiantennus***  **Xiaolong, Yizhou:** gh081, gh084, gh113, gh114, gh115, gh116, gh117, gh124, gh125, gh126, gh127. |
| 12 | ***Fruhstorferiola tonkinensis***  **Gaoji, Sanjiang:** gh041. | 14 | ***Longgenacris_rufiantennus***  **Xiaolong, Yizhou:** gh080. |
