# Supplementary material for: Phylogeny and species delimitation of the genus Longgenacris and Fruhstorferiola viridifemorata species group (Orthoptera: Acrididae: Melanoplinae) based on molecular evidence
Source: PLoS One. 2020 Aug 26;15(8):e0237882. doi: 10.1371/journal.pone.0237882 (PMC7449498; doi:10.1371/journal.pone.0237882)
Supplement: S10 Table — (DOCX) [file pone.0237882.s010.docx]

**S10 Table. Putative species delineated from COI alignment using GMYC model.**

| GMYC species | Morphospecies/Sample name/locality |
| --- | --- |
| 1 | *Conocephalus longipennis*: gh242-243/Guilin, Guangxi Province |
| 2 | *Apalacris tonkinensis*: gh164-168, gh207-211/Nonggang, Guangxi Province |
| 3 | *Ognevia longipennis*: gl0252-0256/Yangjiaping, Hebei Province |
| 4 | *Paratonkinacris vittifemoralis*: gh045-049, gl0247-0250/Gaozhai, Guangxi Province |
| 5 | *Paratonkinacris vittifemoralis*: gl0251/Gaozhai, Guangxi Province |
| 6 | *Emeiacris maculata*: gl0241-0246/Hengshan, Hunan Province |
| 7 | *Emeiacris maculata*: gh075-079, gh088-092/Emeishan, Sichuan Province |
| 8 | *Tonkinacris sinensis*: gl0257-0261/Yong'an, Guangxi Province |
| 9 | ***Fruhstorferiola kulinga***: gl0110, gl0112/Longmenhe, Xingshan County, Hubei Province |
| 10 | ***Fruhstorferiola omei****:* gh085-087/Emeishan, Sichuan Province  ***Fruhstorferiola viridifemorata****:* gh001-007/Longwangshan, Zhejiang Province |
| 11 | ***Fruhstorferiola kulinga***:  gl0101-0106/Hengshan, Hunan Province  gl0107-0108/Jingshan, Hubei Province  gl0111/Longmenhe, Xingshan County, Hubei Province  gl0113-0115/Gaozhai, Guangxi Province  ***Fruhstorferiola huayinensis***:  gl0095-0100/Haopingshi, Shaanxi Province  gl0227-0231/Nanwutai, Shaanxi Province  gl0232-0234/Huayangchuan, Shaanxi Province  gl0235-0239/Baiyunshan, Henan Province |
| 12 | ***Fruhstorferiola kulinga***:  gl0109/Longmenhe, Xingshan County, Hubei Province  ***Fruhstorferiola huayinensis***:  gl0240/Baiyunshan, Henan Province  ***Fruhstorferiola viridifemorata****:*  gh008/ Longwangshan, Zhejiang Province |
| 13 | ***Fruhstorferiola tonkinensis*:**  gh009-014, gh055-059, gh154-158/Nonggang, Guangxi Province  gh040-044/Sanjiang, Guangxi Province  gl0089-0094/Yong'an, Guangxi Province  ***Longgenacris rufiantennus:***  gh080-084, gh113-117, gh123-127/Yizhou, Guangxi Province |
| 14 | ***Longgenacris maculacarina***:  gh015-019, gh144-148, gh159-163/Nonggang, Guangxi Province |
| 15 | ***Ergatettix dorsiferus*:** gh247/Fangchenggang, Guangxi Province |
