# Supplementary figures and images for: Phylogeny and species delimitation of the genus Longgenacris and Fruhstorferiola viridifemorata species group (Orthoptera: Acrididae: Melanoplinae) based on molecular evidence
Source: PLoS One. 2020 Aug 26;15(8):e0237882. doi: 10.1371/journal.pone.0237882 (PMC7449498; doi:10.1371/journal.pone.0237882)

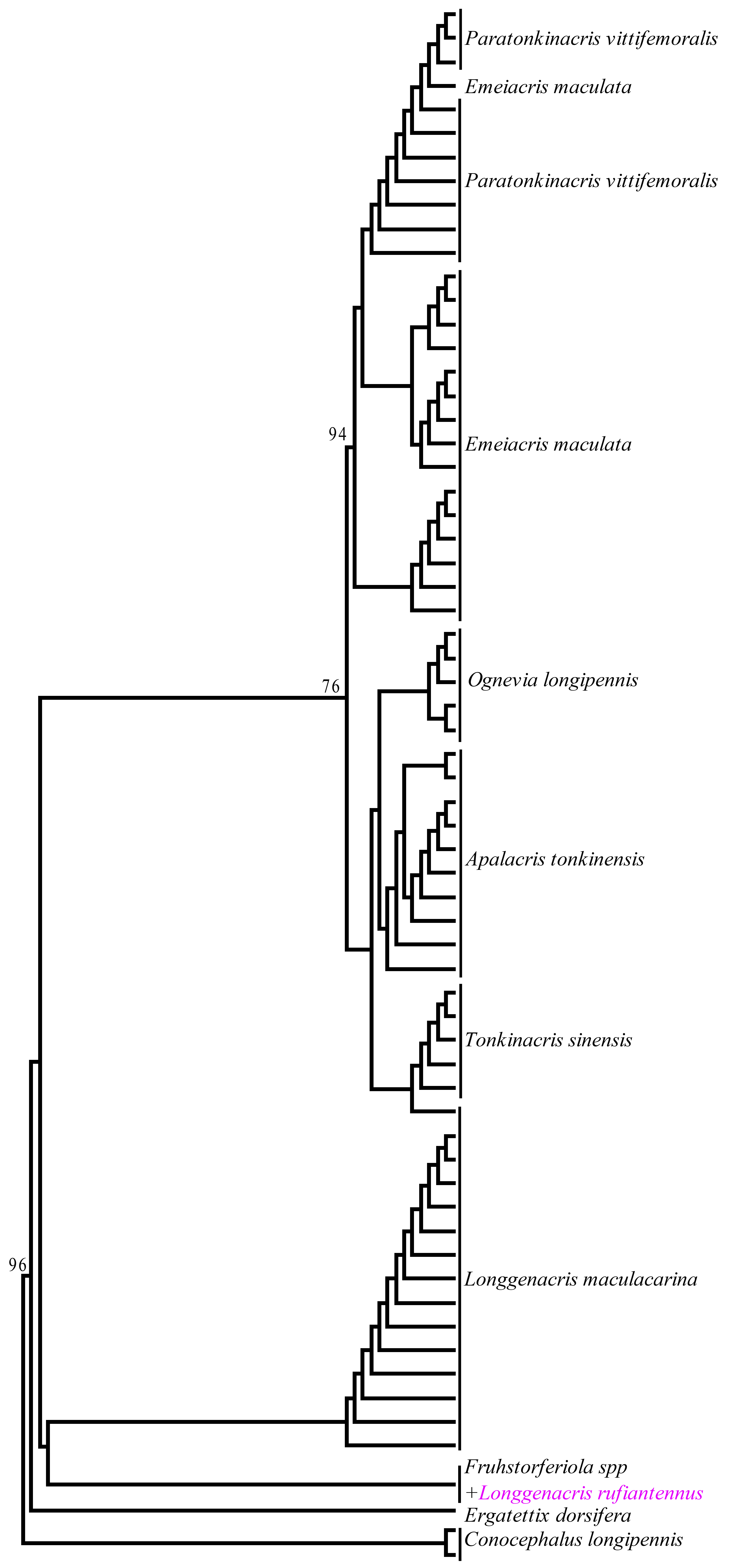


**S1 Fig. Phylogeny deduced in maximum likelihood framework from alignment of ITS1 sequences.**

Supplement: S1 Fig — (DOCX) [file pone.0237882.s011.docx]

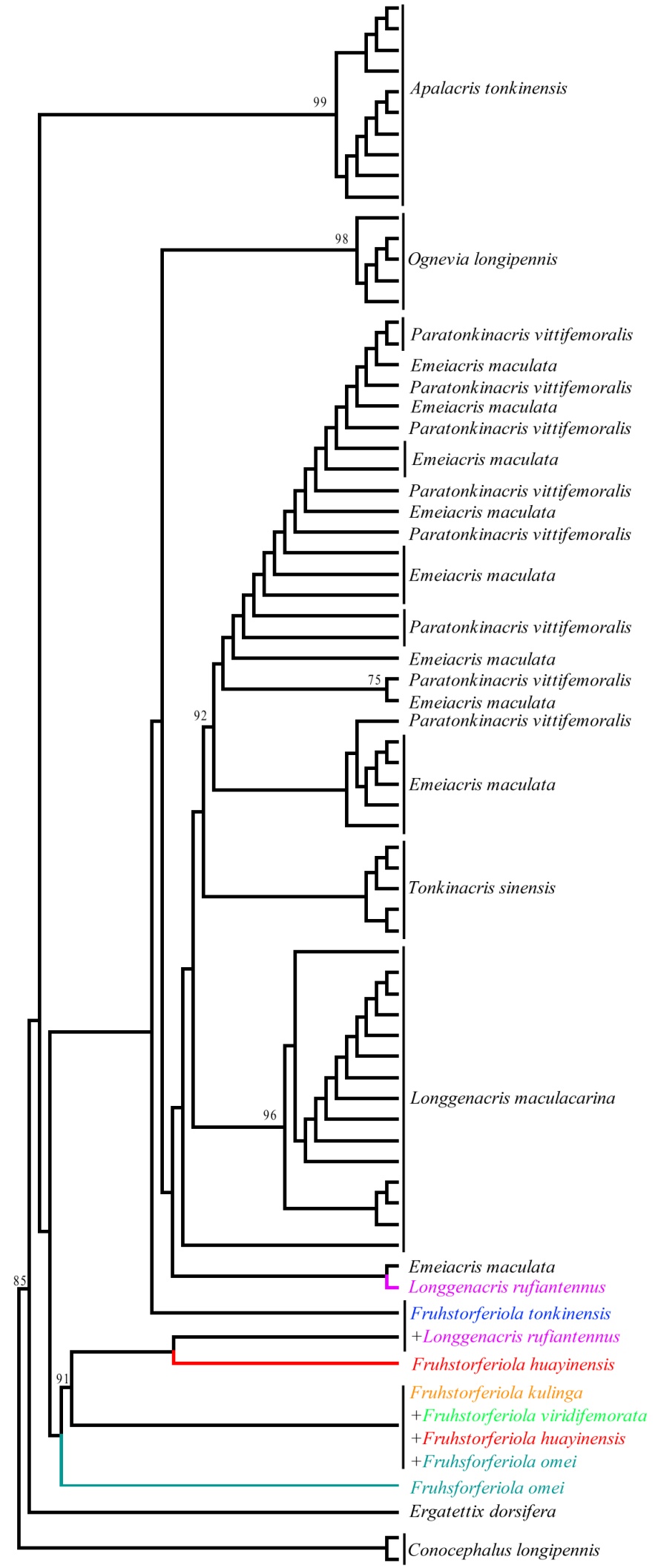


**S2 Fig. Phylogeny deduced in maximum likelihood framework from alignment of ITS2 sequences.**

Supplement: S2 Fig — (DOCX) [file pone.0237882.s012.docx]
