# Supplementary material for: Phylogeny and species delimitation of the genus Longgenacris and Fruhstorferiola viridifemorata species group (Orthoptera: Acrididae: Melanoplinae) based on molecular evidence
Source: PLoS One. 2020 Aug 26;15(8):e0237882. doi: 10.1371/journal.pone.0237882 (PMC7449498; doi:10.1371/journal.pone.0237882)
Supplement: S3 Fig — (DOCX) [file pone.0237882.s013.docx]

**
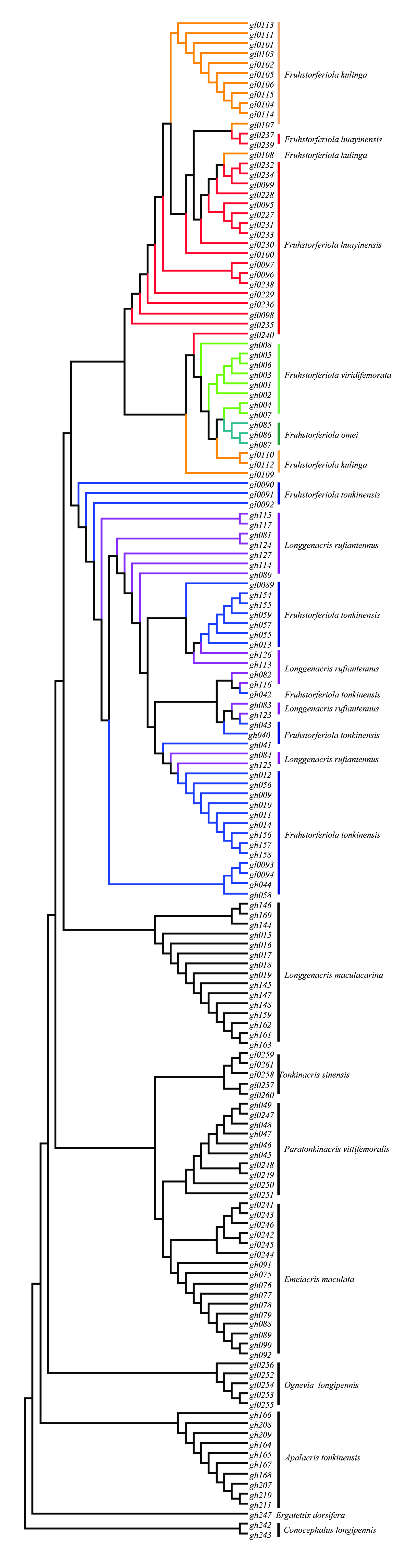

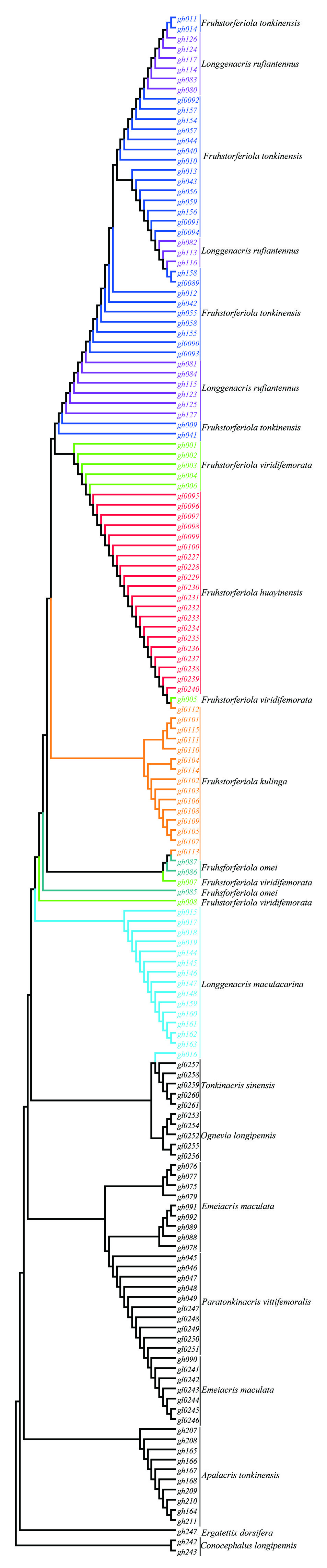
**

**A. tree from COI B. tree from ITS1**

**
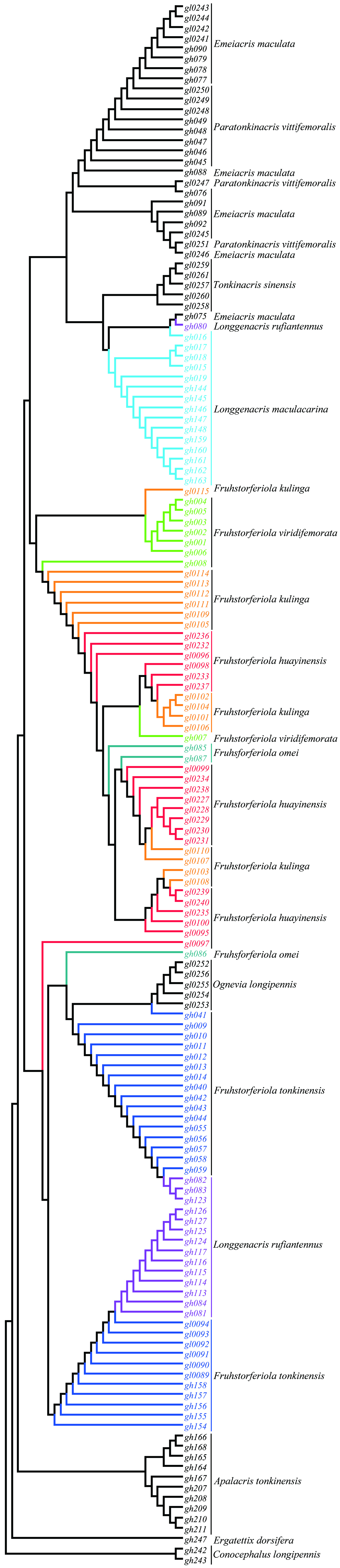

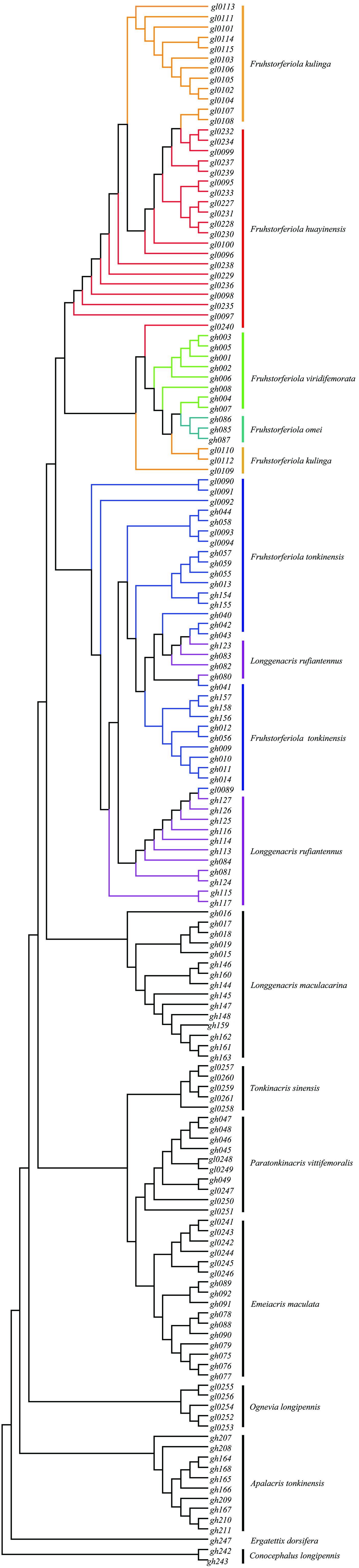
**

**C tree from ITS2 D. tree from combined alignment**

**S3 Fig. NJ trees reconstructed from single and combined alignments of COI, ITS1 and ITS2.** A. NJ tree reconstructed from alignment of COI gene. B. NJ tree reconstructed from alignment of ITS1 sequences. C. NJ tree reconstructed from alignment of ITS2 sequences. D. NJ tree reconstructed from concatenated alignment of COI gene, ITS1 and ITS2 sequences.
