# Supplementary material for: Phylogeny and species delimitation of the genus Longgenacris and Fruhstorferiola viridifemorata species group (Orthoptera: Acrididae: Melanoplinae) based on molecular evidence
Source: PLoS One. 2020 Aug 26;15(8):e0237882. doi: 10.1371/journal.pone.0237882 (PMC7449498; doi:10.1371/journal.pone.0237882)
Supplement: S4 Fig — (DOCX) [file pone.0237882.s014.docx]

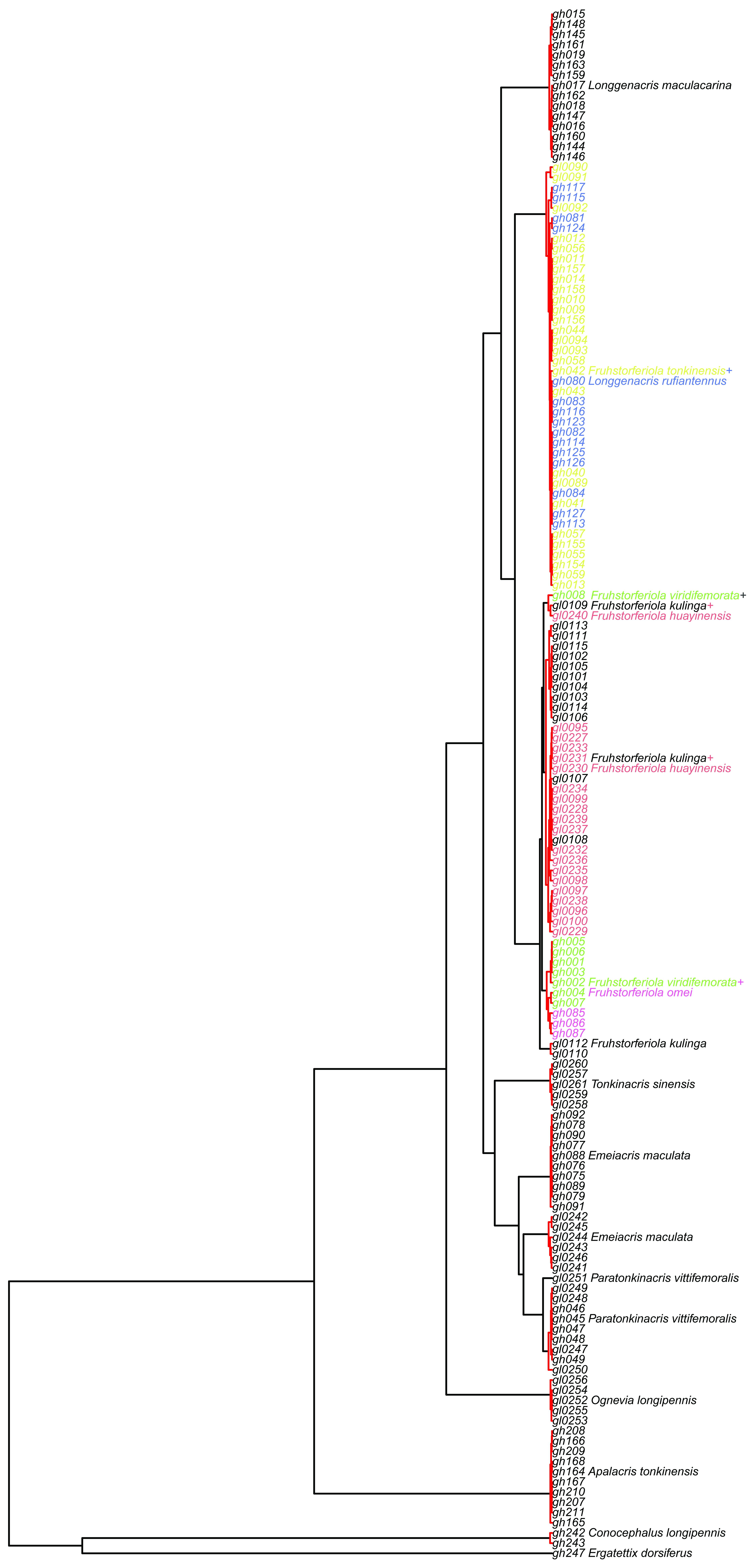


**S4 Fig.** **Species delimitation according to the generalized mixed Yule coalescent (GMYC) single-threshold model using COI data set. The ultrametric tree was obtained in BEAST by setting coalescent prior and strict clock model. Red clusters and black lines (singletons) indicate putative species calculated by the model.**
