# Supplementary material for: Phylogeny and species delimitation of the genus Longgenacris and Fruhstorferiola viridifemorata species group (Orthoptera: Acrididae: Melanoplinae) based on molecular evidence
Source: PLoS One. 2020 Aug 26;15(8):e0237882. doi: 10.1371/journal.pone.0237882 (PMC7449498; doi:10.1371/journal.pone.0237882)
Supplement: Attachment — Submitted filename: PONE-D-20-12129_reviewer.pdf [file pone.0237882.s015.pdf]

PLOS ONE

Phylogeny and species delimitation of the genus Longgenacris and Fruhstorferiola viridifemorata species group (Orthoptera: Acrididae: Melanoplinae) based on molecular evidence

--Manuscript Draft--

**Manuscript Number:**	PONE-D-20-12129
**Article Type:**	Research Article
**Full Title:**	Phylogeny and species delimitation of the genus Longgenacris and Fruhstorferiola viridifemorata species group (Orthoptera: Acrididae: Melanoplinae) based on molecular evidence
**Short Title:**	Species delimitation of Longgenacris and Fruhstorferiola viridifemorata species group
**Corresponding Author:**	Jianhua Huang, Ph.D<br>Central South University of Forestry and Technology<br>Changsha, Hunan CHINA
**Keywords:**	Phylogenetic position; species delimitation; Longgenacris; Longgenacris rufiantennus; Fruhstorferiola viridifemorata species group.
**Abstract:**	Phylogenetic positions of the genus Longgenacris and one of its members, i.e. L. rufiantennus are controversial. The species boundaries of L. rufiantennus + Fruhstorferiola tonkinensis and F. viridifemorata species groups are unclear. In this sutdy, we explored the phylogenetic positions of the genus Longgenacris and the species L. rufiantennus and the relationships among F. viridifemorata group based on the 658-base fragment of the mitochondrial gene cytochrome c oxidase subunit I (COI) barcode and the complete sequences of the internal transcribed spacer regions (ITS1 and ITS2) of the nuclear ribosomal DNA. The phylogenies were reconstructed in maximum likelihood framework using IQ-TREE. K2P distances were used to assess the overlap range between intraspecific variation and interspecific divergence. Phylogenetic species concept and NJ tree, K2P distances, the statistical parsimony network as well as the generalized mixed Yule coalescent model (GMYC) were employed to delimitate the species boundaries in L. rufiantennus and F. viridifemorata species group. The results demonstrated that the genus Longgenacris should be placed in the subfamily Melanoplinae but not Catantopinae, and L. rufiantennus should be a member of the genus Fruhstorferiola but not Longgenacris . Species boundary delimitation confirmed the presence of oversplitting in L. rufiantennus + F. tonkinensis and F. viridifemorata species groups and suggested that each group should be treated as a single species.
**Order of Authors:**	Jingxiao Gu
	Bing Jiang
	Haojie Wang
	Tao Wei
	Liliang Lin
	Yuan Huang
	Jianhua Huang, Ph.D
**Additional Information:**	
**Question**	**Response**
**Financial Disclosure**<br><br>Enter a financial disclosure statement that describes the sources of funding for the work included in this submission. Review the submission guidelines for detailed	This study is supported by the Open foundation for innovation platform of Education Bureau of Hunan Province (18K056) and National Natural Science Foundation of China (No. 31540055, 31260523, 31801993). The funders had no role in study design, data collection and analysis, decision to publish, or preparation of the manuscript.

Powered by Editorial Manager® and ProduXion Manager® from Aries Systems Corporation

requirements. View published research articles from *PLOS ONE* for specific examples.<br><br>This statement is required for submission and **will appear in the published article** if the submission is accepted. Please make sure it is accurate.<br><br>**Unfunded studies**<br>Enter: *The author(s) received no specific funding for this work.*<br><br>**Funded studies**<br>Enter a statement with the following details:<br>• Initials of the authors who received each award<br>• Grant numbers awarded to each author<br>• The full name of each funder<br>• URL of each funder website<br>• Did the sponsors or funders play any role in the study design, data collection and analysis, decision to publish, or preparation of the manuscript?<br>• **NO** - Include this sentence at the end of your statement: *The funders had no role in study design, data collection and analysis, decision to publish, or preparation of the manuscript.*<br>• **YES** - Specify the role(s) played.<br><br>* typeset	
**Competing Interests**<br><br>Use the instructions below to enter a competing interest statement for this submission. On behalf of all authors, disclose any competing interests that could be perceived to bias this work—acknowledging all financial support and any other relevant financial or non-financial competing interests.<br><br>This statement **will appear in the published article** if the submission is accepted. Please make sure it is accurate. View published research articles from *PLOS ONE* for specific examples.	The authors have declared that no competing interests exist.

**NO authors have competing interests**<br><br>Enter: *The authors have declared that no competing interests exist.*<br><br>**Authors with competing interests**<br><br>Enter competing interest details beginning with this statement:<br><br>*I have read the journal's policy and the authors of this manuscript have the following competing interests: [insert competing interests here]*<br><br>* typeset	
**Ethics Statement**<br><br>Enter an ethics statement for this submission. This statement is required if the study involved:<br><br>• Human participants<br>• Human specimens or tissue<br>• Vertebrate animals or cephalopods<br>• Vertebrate embryos or tissues<br>• Field research<br><br>Write "N/A" if the submission does not require an ethics statement.<br><br>General guidance is provided below. Consult the submission guidelines for detailed instructions. **Make sure that all information entered here is included in the Methods section of the manuscript.**	N/A

Powered by Editorial Manager® and ProduXion Manager® from Aries Systems Corporation

**Format for specific study types**<br><br>**Human Subject Research (involving human participants and/or tissue)**<br>• Give the name of the institutional review board or ethics committee that approved the study<br>• Include the approval number and/or a statement indicating approval of this research<br>• Indicate the form of consent obtained (written/oral) or the reason that consent was not obtained (e.g. the data were analyzed anonymously)<br><br>**Animal Research (involving vertebrate animals, embryos or tissues)**<br>• Provide the name of the Institutional Animal Care and Use Committee (IACUC) or other relevant ethics board that reviewed the study protocol, and indicate whether they approved this research or granted a formal waiver of ethical approval<br>• Include an approval number if one was obtained<br>• If the study involved *non-human primates*, add *additional details* about animal welfare and steps taken to ameliorate suffering<br>• If anesthesia, euthanasia, or any kind of animal sacrifice is part of the study, include briefly which substances and/or methods were applied<br><br>**Field Research**<br><br>Include the following details if this study involves the collection of plant, animal, or other materials from a natural setting:<br>• Field permit number<br>• Name of the institution or relevant body that granted permission	
**Data Availability**<br><br>Authors are required to make all data underlying the findings described fully available, without restriction, and from the time of publication. PLOS allows rare exceptions to address legal and ethical concerns. See the PLOS Data Policy and FAQ for detailed information.	Yes - all data are fully available without restriction

A Data Availability Statement describing where the data can be found is required at submission. Your answers to this question constitute the Data Availability Statement and **will be published in the article**, if accepted.<br><br>**Important:** Stating 'data available on request from the author' is not sufficient. If your data are only available upon request, select 'No' for the first question and explain your exceptional situation in the text box.<br><br>Do the authors confirm that all data underlying the findings described in their manuscript are fully available without restriction?	
**Describe where the data may be found in full sentences. If you are copying our sample text, replace any instances of XXX with the appropriate details.**<br><br>• If the data are **held or will be held in a public repository**, include URLs, accession numbers or DOIs. If this information will only be available after acceptance, indicate this by ticking the box below. For example: *All XXX files are available from the XXX database (accession number(s) XXX, XXX.*).<br>• If the data are all contained **within the manuscript and/or Supporting Information files**, enter the following: *All relevant data are within the manuscript and its Supporting Information files.*<br>• If neither of these applies but you are able to provide **details of access elsewhere**, with or without limitations, please do so. For example:<br><br>*Data cannot be shared publicly because of [XXX]. Data are available from the XXX Institutional Data Access / Ethics Committee (contact via XXX) for researchers who meet the criteria for access to confidential data.*<br><br>*The data underlying the results presented in the study are available from (include the name of the third party*	All new haplotype nucleotide sequences are available from the GenBank dtabase (accession numbers are given in S2 Table).

and contact information or URL).<br>• This text is appropriate if the data are owned by a third party and authors do not have permission to share the data.<br><br>* typeset	
Additional data availability information:	

Manuscript
Click here to download Manuscript Gu-2020-manuscript-to plosone.docx

Phylogeny and species delimitation of the genus *Longgenacris* and *Fruhstorferiola viridifemorata* species group (Orthoptera: Acrididae: Melanoplinae) based on molecular evidence

Jingxiao Gu[1,2,#], Bing Jiang[1,2,#], Haojie Wang[3], Tao Wei[4], Liliang Lin[5], Yuan Huang[5], Jianhua Huang[1,2,*]

[1]Key Laboratory of Insect Evolution and Pest Management for Higher Education in Hunan Province, Central South University of Forestry and Technology, Changsha, Hunan 410004, People's Republic of China.
[2]Key Laboratory of Cultivation and Protection for Non–Wood Forest Trees (Central South University of Forestry and Technology), Ministry of Education, Changsha, Hunan 410004, People's Republic of China.
[3]Center for Computational Biology, College of Biological Sciences and Technology, Beijing Forestry University, Beijing 100083, People's Republic of China.
[4]Tanxi Street Agency, Liunan Subdistrict, Liuzhou, Guangxi 545007, People's Republic of China.
[5]College of Life Sciences, Shaanxi Normal University, Xi'an, Shaanxi 710119,People's Republic of China。
[#]They have the equal contribution to the manuscript.
[*]Corresponding author. E-mail: caniscn@aliyun.com.

Abstract. Phylogenetic positions of the genus *Longgenacris* and one of its members, i.e. *L. rufiantennus* are controversial. The species boundaries of *L. rufiantennus*+*Fruhstorferiola tonkinensis* and *F. viridifemorata* species groups are unclear. In this sutdy, we explored the phylogenetic positions of the genus *Longgenacris* and the species *L. rufiantennus* and the relationships among *F. viridifemorata* group based on the 658-base fragment of the mitochondrial gene cytochrome c oxidase subunit I (COI) barcode and the complete sequences of the internal transcribed spacer regions (ITS1 and ITS2) of the nuclear ribosomal DNA. The phylogenies were reconstructed in maximum likelihood framework using IQ-TREE. K2P distances were used to assess the overlap range between intraspecific variation and interspecific divergence. Phylogenetic species concept and NJ tree, K2P distances, the statistical parsimony network as well as the generalized mixed Yule coalescent model (GMYC) were employed to delimitate the species boundaries in *L. rufiantennus* and *F. viridifemorata* species group. The results demonstrated that the genus *Longgenacris* should be placed in the subfamily Melanoplinae but not Catantopinae, and *L. rufiantennus* should be a member of the genus *Fruhstorferiola* but not *Longgenacris*. Species boundary delimitation confirmed the presence of oversplitting in *L. rufiantennus*+*F. tonkinensis* and *F. viridifemorata* species groups and suggested that each group should be treated as a single species.

Key words. Phylogenetic position, species delimitation, *Longgenacris*, *Longgenacris rufiantennus*, *Fruhstorferiola viridifemorata* species group.

Introduction

Taxonomy is a process to take or collate decision continually. Any taxonomic decision taken since the inception of zoological nomenclature in 1758 has relevance today, and on into the future, no matter that decision was right or wrong [1]. The process of modern taxonomy can be viewed as a taxonomic circle, and hypothesis established from any information should be tested with other sources of information, i.e. taxonomists must break out of the circle of inference in species delineation work to raise the entity to species status [2].

Cryptic species usually refers to as one of two or more species that are morphologically indistinguishable in adult stage and incapable of interbreeding since most morphospecies were described based on adult types so far. Cryptic species have been detected in some insect groups through molecular evidences and tested with other information such as morphological, geographical, biological and ecological evidences, etc [3–11]. It is clear that genomic information should be an active component of modern taxonomy, and that integration of the "fashionable" molecular approaches with the classical taxonomic approach is a critical component of reconciling both camps [2, 12, 13].

Despite the existence of cryptic species (overlumping), oversplitting may also exist especially in early described species groups because of the lack of type comparison which usually lead to repeated descriptions of the same species as different ones without actual morphological difference [14]. Incorrect assignment of a species in genus or higher levels will also lead to description of the same species as different ones because the comparison can't be made between the most close relatives. In these cases, morphological revision is necessary to confirm the presence of morphological differences among the closely related species. Moreover, other sources of data, including geographical, biological, ecological, reproductive, behavioral and DNA sequence information, should be used to determine species boundary and test species hypotheses [2].

Longgenacris is a grasshopper genus belonging to subfamily Melanoplinae with *L. maculacarina* You & Li, 1983 as type species [15–17]. The second species of the genus, *L. rufiantennus* Zheng & Wei, 2003, was described based on materials from Xiaolong, Yizhou, Guangxi, China [18], but recently transferred to the genus *Fruhstorferiola* Willemse, 1922 and synonymized with *F. tonkinensis* (Willemse, 1921) based on morphological similarity [16].

Fruhstorferiola is also a genus in Melanoplinae with 13 known species worldwide [17]. According to the shape of cercus, *Fruhstorferiola* can be tentatively divided into three species groups: (1) *F. viridifemorata* group, with cercus of male laterally compressed and expanded into boot-shape apically (Fig 1A), (2) *F. tonkinensis* group, with cercus of male not expanded into boot-shape apically but slender and slightly spear-shaped (Fig 1B), and (3) *F. huangshanensis* group, with cercus of male laterally compressed and semiroundly expanded in apical half but not boot-shaped (Fig 1C). Among the 13 known *Fruhstorferiola* species, 7 species distributed in continental China belong to *F. viridifemorata* group, with 4 species, i.e. *F. viridifemorata*, *F. kulinga*, *F. huayinensis* and *F. omei*, having broad distribution and the remaining 3 species, i.e. *F. brachyptera*, *F. rufucorna* and *Fruhstorferiola xuefengshana*, having been recorded only from the type locality. The main morphological characters used to distinguish species in *F. viridifemorata* group from each other are the length of tegmen, the shape of male cercus in apical portion and teeth in the posterior margin of female subgenital plate. However, these

characters vary even among individuals from the same population. For example, specimens of each species collected from the same locality on the same date exhibit similar pattern of variation in tooth length (Fig 2), with median tooth longer than submedian and lateral teeth in some individuals (Figs 2A, C, E and G), but nearly as long as (Figs 2B, D, F and H) or slightly shorter (Fig 2I) than submedian and lateral teeth in other individuals, or with submedian teeth indistinct or even absent in a few individuals (Fig 2J). Therefore, it is difficult to identify specimens of *F. viridifemorata* group using morphological characters only, and frequently the same specimen could be probably recognized as different species by different identifiers.

Species delimitation using molecular data has attracted more and more attention from systematists and taxonomists because of the rapid development of sequencing techniques and bioinformatic methods. There are many successful cases in grasshoppers using molecular evidence for species delimitation so far [5, 6, 8, 9, 11–14, 19–21]. Molecular approaches for species delimitation can be used not only to confirm delimitations proposed by traditional taxonomy [11], but also to delimit new species under an integrative taxonomy framework despite the possibility of oversplitting sometimes [9]. As for the molecular markers, the most frequently employed one is the mitochondrial gene cytochrome c oxidase subunit I (COI, cox1), which was used either alone [9, 14] or together with some other markers [5, 8, 11, 20].

To clarify the phylogenetic position of *L. rufiantennus* and the relationships among *F. viridifemorata* group, we sequenced the 658-base fragment of the 5' end of mitochondrial gene cytochrome c oxidase subunit I (COI, cox1) corresponding to the barcode region for animals [22], and the complete sequences of the internal transcribed spacer regions (ITS1 and ITS2) of the nuclear ribosomal DNA from149 individuals belonging to 7 genera and 12 species in Acrididae, 1 individual in Tetrigidae and 2 individuals in Tettigoniidae. The phylogeny of the species involved was reconstructed from molecular sequence dataset using maximum likelihood method, and the species boundary was delimited using multiple methods, including genetic distance, NJ tree, the haplotype network constructed using the statistical parsimony method [23], and analysis of the generalized mixed Yule coalescent model (GMYC) [24].

Materials and Methods

Taxon sampling

A total of 152 individuals representing 3 families 9 genera and 14 species were sampled (S1 Table). At least five individuals from each population and as many populations as possible of the widespread species were sampled whenever the specimens were available (S1 Table). The sample and type locality of *F. viridifemorata* group were marked as in the map (Fig 3). Species assignation of specimens was performed mainly following Li & Xia's [25] key to species plus geographical information. For example, the specimens from type locality and neighboring places will be assigned to the same species if there is no distinct difference between them. Partial COI sequences were from our previous study (S2 Table) [14]. All specimens were preserved in 100% ethanol and stored at room temperature.

DNA extraction, PCR amplification and sequencing

Whole genomic DNA was extracted from muscle tissue of the hind femur using a routine phenol/chloroform method [26]. The primers for the amplification of COI fragment followed our

previous study: COBU (5'-TYTCAACAAAYCAYAARGATATTGG-3') and COBL (5'-TAAACTTCWGGRTGWCCAAARAATCA-3') [14]. Amplification of complete sequences of ITS1 and ITS2 employed the following primers: 18sF1 (5'-ATGTGCGTTCRAAATGTCGATGTTCA-3') and 5.8sB1d (5'-ATGTGCGTTCRAAATGTCGATGTTCA-3') for ITS1 [27], ITS3 (5'-GCATCGATGAAGAACGCAGC-3') and ITS4(5'-TCCTCCGCTTATTGATATGC-3') for ITS2 [28].

PCRs were carried in a 25 μl reaction mixture containing 13.875 μl of ultrapure water, 2.5 μl of 10×PCR buffer (Mg2+free), 2.5 μl of $MgCl_2$ (25 mM), 2 μl of dNTP (2.5 mM), 1.5 ml of each primer (0.01 mM), 0.125 μl of TaKaRa r-Taq polymerase, and 1 μl of DNA template. The cycling protocol consisted of an initial denaturation step at 95°C for 5 min, followed by 30–35 cycles of denaturation at 94°C for 45 s, annealing at 48°C for 45 s and extension at 70°C for 1 min 30 s, and a final extension at 72°C for10 min and then held at 4°C. PCR products were sent to the biological company and sequenced bidirectionally after purification. Sequencing primers were the same as those for PCR amplification.

Sequence assembly and alignment

Assembly of the raw sequencing files was implemented in the Staden Package [29]. The assembled sequences were aligned using Clustal X [30], and the primer sequences in both ends of the sequences were excised to remove artificial nucleotide similarity derived from PCR amplification. COI nucleotide sequences were translated into amino acid sequences to detect the potential nuclear mitochondrial pseudogenes (numts) based on the presence of premature stop codons and shifts in reading frame [31, 32]. Haplotype nucleotide sequences are deposited in GenBank (MH934098—MH934186, S2 Table). Each haplotype was blasted using MEGABLAST option against the nucleotide collection (nr/nt) available on the NCBI website (http://blast.ncbi.nlm.nih.gov/Blast.cgi?CMD = Web&PAGE_TYPE = BlastHome). Only haplotypes that blasted within the correct suborder with E-values ≤ 1.00E-30 were included in this study [33].The combined data set of COI, ITS1 and ITS2 was concatenated in SequenceMatrix [34].

Intraspecific variation, interspecific divergence and phylogeny reconstruction

Sequence divergences were calculated using the Kimura two parameter (K2P) distance model [35, 36]. The calculation of the sequence divergences was implemented in MEGA7.0 [37].

The phylogenies were reconstructed in maximum likelihood framework with *Ergatettix dorsiferus* in Tetrigidae and *Conocephalus longipennis* in Tettigoniidae as outgroups. Maximum-Likelihood phylogenies were reconstructed using IQ-TREE, a fast and effective stochastic algorithm combining hill-climbing approaches and a stochastic perturbation method [38], best-fit models of nucleotide evolution and best-fit partitioning scheme were selected using ModelFinder [39], the approximately unbiased branch support values were calculated using UFBoot2 [40], and the analysis was performed in W-IQ-TREE using default sets most of the time [41].

To provide a profile for the setup of taxa and groups for calculating genetic distances, a neighbor-joining (NJ) tree of K2P distances was created to provide a graphic representation of the patterning of divergence between species [42] because of its strong track record in the analysis of large species assemblages [43]. NJ tree building with 1000 bootstrap replicates was implemented in

MEGA7 [37].

Network analysis

Statistical parsimony network [23] can provide more significant inferences about evolutionary relationships than traditional bifurcating trees when divergences are low. The 95% parsimony connection limit may be used as an objective standard of genetic differentiation for the identity of traditional species or ESUs [44, 45]. In most of published network analyses, alignments of DNA sequences typically fall apart into a separate subnetwork for each Linnean species (but with a higher rate of true positives for mtDNA data) and DNA sequences from single species typically stick together in a single haplotype network [46]. Therefore, we constructed haplotype networks for *Longgenacris* species and *F. viridifemorata* group. The construction of haplotype networks was implemented in TSC1.21 [47].

Analysis of the generalized mixed Yule coalescent model (GMYC)

The single-threshold GMYC analyses were conducted in R v3.6.1 in a Windows environment with the use of the *splits* package. The ultrametric single-locus gene tree required for the GMYC method was obtained using BEAST 1.8.2 [48] with 10 million MCMC generations under the Yule speciation model. A strict molecular clock was shown to be appropriate to infer the ultrametric trees through the model comparison using a Bayes factor test in Tracer 1.6. Effective sample sizes (ESS) and trace plots estimated with Tracer 1.6 were used as convergence diagnostics, and a burn-in of one million generations was used to avoid suboptimal trees in the final consensus tree.

Results

Phylogeny

Phylogeny of the taxa involved in this study was reconstructed in maximum likelihood framework using separate alignments of COI, ITS1, ITS2 and their concatenated alignment, respectively.

The trees inferred from COI and the combined alignments displayed similar topologies (Fig 4). Nearly all species formed reciprocally monophyletic clades except *F. tonkinensis*+ *L. rufiantennus* and *F. viridifemorata* groups. The main differences between the single COI gene tree and the combined alignment tree were the placements of *Emeiacris maculata*, of which two clades did not form monophyletic clade but were added in turn to the clade of its closest relative *Paratonkinacris vittifemoralis* in COI gene tree (Fig 4A), and *Apalacris tonkinensis*, which is a member of the subfamily Catantopinae but had a closer relationship to most of Melanoplinae members than *Tonkinacris sinensis* in the combined alignment tree (Fig 4D), i.e. Melanoplinae formed a monophyletic clade in COI gene tree but not in the combined alignment tree. For the clade of *F. tonkinensis*+ *L. rufiantennus* group, all of the 15 individuals of *L. rufiantennus* scattered within the clade of *F. tonkinensis* (Figs. 4B and E). Individuals within *F. viridifemorata* group clustered neither by species nor populations (Figs 4C and F).The four individuals of *F. kulinga* from Longmenhe, Xingshan County, Hubei Province exhibited most complicated relationship with other species/populations, with two individuals close to *F. omei* and *F. viridifemorata*, one close to *F. huayinensis*, and the remaining one close to individuals of *F. kulinga* from Hunan and Guangxi poppulations in tree from combined dataset or located at the base of the tree from COI gene (Figs

4C and F). *L maculacarina* consistently formed a monophyletic clade and had a most close relationship to *Fruhstorferiola* species (Figs 4A and D).

The tree inferred from ITS1 sequences had less resolution at species level (S1 Fig). Although *F. tonkinensis*+ *L. rufiantennus* group formed a monophyletic clade, but it falled into the clade of *F. viridifemorata* group and all *Fruhstorferiola* species formed a larger monophyletic clade. *P. vittifemoralis* and *E. maculata* formed a large monophyletic clade together, but neither of them formed monophyletic subclade. The remaining four distantly related species formed monophyletic clades each, except one individual of *L maculacarina* falled into the clade of *T. sinensis*. *Apalacris tonkinensis* falled into the members of the subfamily Melanoplinae just as in the combined alignment tree. The tree inferred from ITS2 sequences had a similar topology to that from ITS1 sequences, but all members of the tribe Melanoplinae formed a large monophyletic clade as in COI gene tree (S2 Fig).

As for NJ trees, the one deduced from single COI gene (S3 Fig A) had extremely similar topology to that of ML tree. Monophyletic clades could be retrieved consistently for distantly related species and closely related species groups both in single and combined alignment trees with exceptions for only a few individuals in single ITS1 and ITS2 alignment trees. For example, individual gh016 of *L. maculacarina* falled ino the clade of *T. sinensis* in the tree from ITS1 sequences (S3 Fig B), individual gh041 of *F. tonkinensis* falled into the clade of *O. longipennis* in the tree from ITS2 sequences, individual gh086 of *F. omei*, gl0097 of *F. huayinensis*, gh075 of *E. maculata* and gh080 of *L. rufiantennus* escaped from their own stem clades respectively (S3 Fig C). Monophyly of Melanoplinae was supported in both single and combined alignment trees (S3 Fig A–D).

Intraspecific variation and interspecific divergence

Based on the neighbor-joining (NJ) tree of K2P distances, taxa or groups were set up to calculate the intraspecific variations and interspecific divergences. The results showed that, for COI sequences, variations within population were mostly distinctly less or slightly larger than 1%, except those of *F. kulinga* within Longmenhe population, of which the maximum pairwise distance was 2.33%; intraspecific variations between populations were usually less than 3% (S3 Table), a putative threshold for species assignment proposed by previous study (Herbert *et al.*, 2003), with *E. maculata* as the single exception which had much higher intraspecific variation(4.24–4.73%, average 4.45%) between interpopulation individuals. Two populations of *E. maculata*, one from Hengshan of Hunan and the other from Gaozhai of Guangxi, were sampled; the variations within population were less than 1% but those between populations ranged from 4.24% to 4.73%. Both ITS1 and ITS2 sequences showed much lower intraspecific variations but had similar distribution pattern (S3 Table).

The interspecific divergences of COI sequences within *F. viridifemorata* groups ranged from 1.00% to 2.03%, those between species of *F. viridifemorata* groups and *F. tonkinensis* were up to 5.53-6.08% and the one between *F. tonkinensis* and *L. rufiantennus* was 0.33%, but that between *L. rufiantennus* and *L. maculacarina* was as high as 7.33% (S4 Table). The interspecific divergences calculated from ITS1 and ITS2 sequences displayed similar distribution patterns (S5, S6 Tables), i.e. species within *F. viridifemorata* group and *F. tonkinensis*+ *L. rufiantennus* group had much lower between-species mean distances but the mean distances between other pairewise species were distinctly much higher. For all of three alignments, the distances between species within

Melanoplinae were constantly lower than those between species in Melanoplinae and that out of Melanoplinae (Tables S4–S6).

Species boundary delimitation

(1) *Fruhstorferiola tonkinensis* + *Longgenacris rufiantennus* group

Considering the high similarity between *F. tonkinensis* and *L. rufiantennus*, we sampled 15 individuals of *L. rufiantennus* from its type locality, 27 individuals of *F. tonkinensis* in total from five populations and 15 individuals of *L. maculacarina* for comparison. The results showed that *L. maculacarina* usually formed a monophyletic clade, but all individuals of *L. rufiantennus* fell completely into the clade of *F. tonkinensis* in NJ trees reconstructed both from single and combined alignment sequences (Figs 5A, S3 Figs A–D), with only one exceptive individual for each species escaping from its own stem clade in NJ tree of ITS2 sequences, i.e. individual gh080 of *L. rufiantennus* clustered into a clade together with gh075 of *E. maculata* and individual gh016 of *L. maculacarina*, and individual gh041 of *F. tonkinensis* falled into the clade of *O. longipennis* (S3 Fig C).

For COI sequences, mean intraspecific variations within each species were all distinctly less than 1% (Table 1). Pairwise intraspecific variations within *F. tonkinensis* ranged from 0 to 1.08%, and that within *L. rufiantennus* ranged from 0 to 0.46%. Pairwise interspecific divergence between *F. tonkinensis* and *L. rufiantennus* ranged from 0 to 0.77%, and completely fell into the range of pairwise intraspecific variations within *F. tonkinensis*. Pairwise interspecific divergence between *L. rufiantennus* and *L. maculacarina* ranged from 7.24-7.92% and the mean divergence was 7.33% (Table 1). For ITS1 and ITS2 sequences, both intraspecific variations and interspecific divergences were much lower but had similar variation patterns (S3, S5, S6 Tables).

Table 1. Intraspecific variation and interspecific divergence of *F. tonkinensis*+*L. rufiantennus* group and *L. maculacarina* calculated from COI sequence.

Species	Intraspecific variation	Interspecific divergence (Pairwise/mean)	
	(Pairwise/mean)	*F. tonkinensis*	*L. rufiantennus*
F. tonkinensis	0-1.08% (0.45%)	----	
L. rufiantennus	0-0.46% (0.11%)	0 to 0.77%(0.33%)	
L. maculacarina	0-0.61% (0.17%)	6.72-7.93% (7.40%)	7.24-7.92% (7.33%)

Analysis with haplotype network led to a similar result. The numbers of COI haplotypes detected in *F. tonkinensis*, *L. rufiantennus* and *L. maculacarina* were 12, 3 and 4, respectively (S7 Table). Among the 3 haplotypes detected in *L. rufiantennus*, the one represented by 11 individuals was shared with *F. tonkinensis*, and the other two represented each by a single individual were private for *L. rufiantennus*. In the net work from COI haplotypes (Fig 5B), haplotypes of *L. maculacarina* formed a separate clade, and those of *F. tonkinensis* and *L. rufiantennus* formed another clade together. In the clade of *F. tonkinensis*+*L. rufiantennus*, no haplotypes from the same population formed monophyletic subclade. For ITS1 sequences, only 5 haplotypes were detected in *F. tonkinensis* and all individuals of *L. rufiantennus* shared the same haplotype with some individuals of *F. tonkinensis* from the 5 sampled populations (S8 Table). In the net work from ITS1 sequences (Fig 5C), haplotypes of *F. tonkinensis* and *L. rufiantennus* formed a clade but no

monophyletic subclade, and the 2 haplotypes of *L. maculacarina* did not connect into a single net work, but separated from each other. For ITS2 sequences, 3 haplotypes were detected for *F. tonkinensis* and *L. rufiantennus* each, with two shared haplotypes (S9 Table). Haplotypes of all 3 species connected into a single net work together with haplotypes of *F. viridifemorata* group (Fig 5D), indicating a much lower evolution rate in ITS2 sequence.

In GMYC analysis based on COI sequences, 14 putative species were delineated from the whole data set (S10 Table, S4 Fig). The 10 individuals of *Paratonkinacris vittifemoralis* collected from the same locality (Gaozhai, Maoershan, Xing'an county, Guangxi) were delineated into 2 putative species, one represented by 9 individuals and the other by the single sample gl0251. Each population of *Emeiacris maculata* was delineated as an independent species. *F. tonkinensis* and *L. rufiantennus* were delineated as the same species (Figs6C, S4 Fig; S10 Table). Samples of *F. viridifemorata* group were delineated into 4 putative species by neither morphospecies nor populations, and samples of each remaining species were delineated as an independent species.

(2) *Fruhstorferiola viridifemorata* group

In an earlier study, the relationship between *F. kulinga* and *F. huayinensis* was discussed using single COI barcoding fragment, and the result did not support the validity of *F. huayinensis* [14]. Not only *F. kulinga* and *F. huayinensis* are difficult to distinguish morphologically, but also the other 5 species belonging to *F. viridifemorata* group display nearly no distinguishable morphological difference from each other. Therefore, we include *F. viridifemorata* and *F. omei* into the present analysis to explore the relationship among them again.

In the NJ tree of COI sequences, the four species of *F. viridifemorata* group formed a monophyletic clade. Although the 3 individuals of *F. omei* formed a so-called monophyletic subclade, but it completely fell into the larger subclade of *F. viridifemorata*. Individuals of the other 3 species clustered neither by species nor populations (Fig7A). In the NJ tree of ITS1 sequences, species of *F. viridifemorata* group did not form a monophyletic clade, but formed three separate clades and added in turn to the clade of *F. tonkinensis*+*L. rufiantennus* group together with one individual of *F. omei* and one of *F. viridifemorata* (S3 Fig B). In the NJ tree of ITS2 sequences, most individuals of *F. viridifemorata* group formed a monophyletic clade, but again clustered neither by species nor populations, except individual gh086 of *F. omei* which clustered with a subclade of *O. longipennis*+*F. tonkinensis*+*L. rufiantennus*, and individual gl0097 of *F. huayinensis* which clustered with the larger subclade of *O. longipennis*+*F. tonkinensis*+*L. rufiantennus*+gh086 (S3 Fig C).

Mean intraspecific variations within each species calculated from COI sequences were distinctly less or slightly larger than 1%, and the largest pairwise intraspecific variation was as high as 2.97% in *F. kulinga*, but still slightly less than 3%. Broad overlaps between intraspecific genetic variations and interspecific divergences are found in all species pairs (Table 2). For ITS1 and ITS2 sequences, all intraspecific variations within population are distinctly less than 1% and only a few ones between populations are slightly more than 1% (S3 Table). As for the interspecific divergences, the genetic distances between species within the genus *Fruhstorferiola* are all less than 1%, and those between *Fruhstorferiola* species and the species in other genera are distinctly more than 2% (S5, S6 Tables).

Table 2. Intraspecific variation and interspecific divergence of *F. viridifemorata* group calculated from COI

sequence.

Species	Intraspecific variation (Pairwise/mean)	Interspecific divergence (Pairwise/mean)		
		F. viridifemorata	*F. omei*	*F. huayinensis*
F. viridifemorata	0-1.23% (0.60%)	----	----	----
F. omei	0.15-0.77% (0.51%)	0.46-1.54% (1.00%)	----	----
F. huayinensis	0-1.85% (0.57%)	0.61-2.48% (1.91%)	1.23-2.80% (2.03%)	----
F. kulinga	0-2.97% (1.02%)	0.46-2.32% (1.48%)	0.92-2.64% (1.65%)	0.15-2.96% (1.12%)

Haplotype network analysis detected no shared haplotype in COI sequences among the four species (S7 Table), but shared haplotypes occur in ITS1 and ITS2 sequences among these species (S8, S9 Tables). In the network from COI haplotypes (Fig 7B), all haplotypes are connected into a large network in a maximum connection steps of 11 at 95%, but three of the four species do not form reciprocally monophyletic clades. Although the three haplotypes of *F. omei* forms a so-called monophyletic clades, the maximum mutational steps of haplotypes within *F. omei* reaches 4 steps, slightly higher than the minimum mutational steps of haplotypes between *F. omei* and *F. viridifemorata*. For ITS1 sequences, a haplotype shared by three species with high frequencies as well as another one shared by two species with low frequencies are found (S8 Table). In the network from ITS1 haplotypes (Fig 7C), still no species forms reciprocally monophyletic clades. For IIS2 sequences, a haplotype shared by four species is found (S9 Table) and all haplotypes of *F. viridifemorata* group and *F. tonkinensis*+*L. rufiantennus* group are connected into a single net work as mentioned in the previous section (Fig 5D).

For the four putative species delineated in GMYC analysis (S10 Table), the putative species 9 consists of two of the four individuals of *F. kulinga* from Longmenhe, Hubei Province, the putative species 10 consists of all three individuals of *F. omei* from Emeishan, Sichuan Province and seven of the eight individuals of *F. viridifemorata* from Longwangshan, Zhejiang Province, the putative species 11 consists of most individuals of *F. kulinga* and *F. huayinensis* from different localities, the putative species 12 consists of one individual of each species of *F. kulinga* and *F. huayinensis* and *F. viridifemorata*. Three of the four putative species, each consisting of individuals from vast area, contain individuals of at least two morphospecies, and individuals in three populations (Baiyunshan, Longmenhe, Longwangshan) were assigned to at least 2 GMYC species.

Discussion

Phylogenetic position and species delimitation of *Longgenacris rufiantennus*

Although being placed in the genus *Longgenacris* originally, *L. rufiantennus* has substantial differences from its congener *L. maculacarina* concerning the length of tegmina and wings, the shape of cerci in male, the subgenital plate in female as well as the structure of male genitalia, and shows no morphological difference from *F. tonkinensis* [16]. Phylogeny reconstructed from different datasets consistently supported the closer relationship of *L. rufiantennus* with *F. tonkinensis,* and *L. maculacarina* usually formed an independent monophyletic clade as a sister group of the genus *Fruhstorferiola* (Figs 4A and D, S1 Fig, S2 Fig). Therefore, *L. rufiantennus* should be regarded as a member of the genus *Fruhstorferiola* but not a member of *Longgenacris* no matter according to morphological or molecular evidences.

As for the relationship between *L. rufiantennus* and *F. tonkinensis*, all analysis (NJ tree,

genetic distance and haplotype network) lead to the same result that they should be the same species but not two independent species because all individuals of *L. rufiantennus* fall into the clade of *F. tonkinensis* in NJ trees (Fig 5A, S3 Fig), the pairwise genetic distances within *F. tonkinensis* completely overlapped with those between *F. tonkinensis* and *L. rufiantennus* (Table 1), the COI haplotype of *L. rufiantennus* with highest frequency are shared with *F. tonkinensis* (S7 Table) and all haplotypes of the two species formed a whole network under the 95% parsimony connection limit (Fig 5B) , GMYC analysis delineated them as the same species (Fig 6C, S4 Fig; S9 Table). Therefore, this study confirmed the synonymy of *L. rufiantennus* with *F. tonkinensis* [16].

Subfamily placement of the genus *Longgenacris*

The genus *Longgenacris* was originally placed in the subfamily Melanoplinae and considered most similar to the genus *Ognevia* Ikonnikov, 1911 [15]. The phylogenetic position of the genus was discussed recently based on morphological characters because once it was regarded as a member of the subfamily Catantopinae [16]. In this study, the genus *Longgenacris* consistently has the most close relationship with and is most of the time the sister group of the genus *Fruhstorferiola* (Figs 4A, D, S1–S3 Figs). Therefore, this study support the original placement of the genus *Longgenacris* in the subfamily Melanoplinae.

Species delimitation of *Fruhstorferiola viridifemorata* group

To explore the species boundary among species in *F. viridifemorata* group in a larger scale than the previous study [14], samples of two additional species, i.e. *F. viridifemorata* and *F. omei*, were added to the present study, and ITS region was employed in addition to COI sequence. However, the increases of the sampled species and molecular markers did not lead to different result from that of previous study [14]. It seemed that the resolution of the datasets were contributed mainly by COI gene sequences, and ITS region had a much lower evolution rate than COI gene in our datasets. No matter the non-monophyly of the morphospecies in NJ trees, the extent of the overlaps between pairwise intraspecific genetic variations and interspecific divergences, or the haplotype networks, all results did not support the validity of the four independent morphospecies, and this was consistent with the results of our morphological recomparison mentioned in introduction section. As for the result of GMYC analysis, we will discuss it in detail in the following section.

Cryptic species or genetic polymorphism: testing species hypotheses with diagnostic characters from different approaches

In the case of *L. rufiantennus*, a comprehensive comparison across members of closely related genera revealed high morphological similarity between *L. rufiantennus* and *F. tonkinensis*, and a synonymy was proposed based on morphological evidences [16]. This decision is confirmed by molecular evidences again in this study, resulting in a perfect synergy of resolution that an integrated taxonomy is capable of attaining [2].

In the case of *F. viridifemorata* group, the condition is a little more complicated. Although NJ tree, pairwise genetic distances and haplotype networks retrieved coincident results corresponding to the result of morphological recomparison, the GMYC analysis of COI gene delineated four molecular operational taxonomic units (MOTUs) from samples of *F. viridifemorata* group (Fig 6D, S4 Fig; S10 Table). Do the four MOTUs represent morphologically cryptic species or only ancient

genetic polymorphism? Among species of *F. viridifemorata* group, the morphological characters originally employed to describe the different species have been approved to be variable even within populations of the same species (Fig 2), and most analyses of molecular evidences are congruent with the result of morphological reexamination. As for the four MOUTs delineated by GMYC analysis using COI gene (S10 Table), they didn't be supported by either morphologocial or geographical informations. Furthermore, this approach tends to overestimate the number of species because of errors in reconstruction of ultrametric input trees [49, 50], or in the presence of high population structure or considerably high values of effective population size [51, 52], especially when mitochondrial genomic dataset is employed [11]. Although GMYC was considered a robust tool for delimiting species when only single-locus information is available [53], it cannot be used as sufficient evidence for evaluating the specific status of particular cases without additional data [54]. Therefore, we can't be able to break out of the taxonomic circule at present, and prefer to consider the four MOTUs of *F. viridifemorata* group delineated with GMYC model as ancient genetic polymorphism. The diverse and complicated relationships of Longmenhe population of *F. kulinga* with other species (Figs 4C and F) indicates the possibility that Longmenhe population has the highest genetic diversity and might be a centre of dispersal for a widespread species. This molecular study will serve as a robust basis to carry out further studies using additional molecular markers and morphological information from different character systems.

Although we increased the numbers of sampled species and molecular markers in this study, a sample size of three individuals for *F. omei* was a little insufficient, no individual from type localities was sampled for *F. viridifemorata* and *F. kulinga*, and molecular markers employed were still not enough. Considering the genomic features of species complex in early stage of parallel speciation or divergence where conflicting inferences are more prone to appear [7, 13], the discordant pattern between mitochondrial and nuclear DNA [19, 55, 56], and the possibility of the concurrence of both cryptic species and morphological polymorphism in the same group [57], a more comprehensive study combining complete mitochondrial genome, more nuclear genes and morphological data is going to carry out. Anyway, the consensus of numerous independent criteria is needed to define species boundaries, particularly in cases of recent speciation events or species that are very similar and difficult to distinguish morphologically [9, 58]. We believe that a more unambiguous outline of the relationship within *F. viridifemorata* group will be achieved with the accumulation of more types of information.

Supporting Information

S1 Table. Materials involved in this study.

S2 Table. Mapping table between GenBank accession numbers and voucher numbers.

S3 Table. Intraspecific variations calculated from different datasets.

S4 Table. Mean genetic distances between species calculated from COI alignment.

S5 Table. Mean genetic distances between species calculated from ITS1 alignment.

S6 Table. Mean genetic distances between species calculated from ITS2 alignment.

S7 Table. Haplotyptes of COI detected from samples of *F. viridifemorata* and *F. tontinensis*+*L. rufiantennus* groups

S8 Table. Haplotyptes of ITS1 detected from samples of *F. viridifemorata* and *F. tontinensis*+*L. rufiantennus* groups

S9 Table. Haplotyptes of ITS2 detected from samples of *F. viridifemorata* and *F. tontinensis+L. rufiantennus* groups

S10 Table. Putative species delineated from COI alignment using GMYC model.

S1 Fig. Phylogeny deduced in maximum likelihood framework from alignment of ITS1 sequences.

S2 Fig. Phylogeny deduced in maximum likelihood framework from alignment of ITS2sequences.

S3 Fig. NJ trees reconstructed from single and combined alignments of COI, ITS1 and ITS2.

S 4 Fig. Species delimitation according to the generalized mixed Yule coalescent (GMYC) single-threshold model using COI data set.

Acknowledgement.

We would like to thank Mr. Tao Wang for his help in implementing GMYC analysis, to thank Dr. Xiaolong Lin for his comments on species delimitation methods, to thank Dr. Xun Bian for his help in drawing the map in R. This study was supported by the Open foundation for innovation platform of Education Bureau of Hunan Province (18K056) and National Natural Science Foundation of China (No. 31540055, 31260523, 31801993).

References

1. Bolton B. A new general catalogue of the ants of the world. Cambridge, Mass.: Harvard University Press; 1995.
2. DeSalle, R, Egan MG, Siddall M. The unholy trinity: taxonomy, species delimitation and DNA barcoding. Philos. T. R. Soc. B. 2005; 360: 1905–1916.
3. Hebert PDN, Penton EH, Burns JM, Janzen DH, Hallwachs W. Ten species in one: DNA barcoding reveals cryptic species in the neotropical skipper butterfly *Astraptes fulgerator*. P. Natl. Acad. Sci. U.S.A. 2004; 101(41): 14812–14817.
4. Smith MA, Woodley NE, Janzen DH, Hallwachs W, Hebert PDN. DNA barcodes reveal cryptic host-specificity within the presumed polyphagous members of a genus of parasitoid flies (Diptera: Tachinidae). P. Natl. Acad. Sci. U.S.A. 2006; 103: 3657–3662.
5. López H, Contreras-Díaz HG, Oromí P, Juan C. Delimiting species boundaries for endangered Canary Island grasshoppers based on DNA sequence data. Conserv. Genet. 2007; 8: 587–598.
6. López H, Hernández-Teixidor D, Macías-Hernández N, Juan C, Oromí, P. A taxonomic revision and species delimitation of the genus *Purpuraria* Enderlein, 1929 (Orthoptera: Pamphagidae) using an integrative approach. J. Zool. Syst. Evol. Res. 2013; 51(3): 173-186.
7. Soria-Carrasco V, Gompert Z, Comeault AA, Farkas TE, Parchman TL, Johnston JS, et al. Stick insect genomes reveal natural selection's role in parallel speciation. Science. 2014; 344: 738–742.
8. Pedraza-Lara C, Barrientos-Lozano L, Rocha-Sánchez AY, Zaldívar-Riverón A. Montane and coastal species diversification in the economically important Mexican grasshopper genus *Sphenarium* (Orthoptera: Pyrgomorphidae). Mol. Phylogenet. Evol. 2015; 84: 220–231.
9. Pocco ME, Minutolo C, Dinghi PA, Lange CE, Confalonieri VA, Cigliano MM. Species delimitation in the Andean grasshopper genus *Orotettix* Ronderos and Carbonell (Orthoptera: Melanoplinae): an integrative approach combining morphological, molecular and biogeographical data. Zool. J. Linn. Soc.-Lond. 2015; 174(4): 733–759.

10. Tyagi K, Kumar V, Singha D, Chandra K, Laskar BA, Kundu S, et al. DNA Barcoding studies on Thrips in India: cryptic species and species complexes. Sci. Rep-UK. 2016; 7: 4898.
11. Jesús-Bonilla VS, Barrientos-Lozano L, ZaldÍvar-RiverÓn A. Sequence-based species delineation and molecular phylogenetics of the sitional Nearctic-Neotropical grasshopper genus *Taeniopoda* (Orthoptera, Romaleidae). Syst. Biodivers. 2017; 15(6): 600–617.
12. Solís-Lemus C, Knowles LL, Ańe Ć. Bayesian species delimitation combining multiple genes and traits in a unified framework. Evolution. 2015; 69(2): 492–507.
13. Noguerales V, Cordero PJ, Ortego J. Integrating genomic and phenotypic data to evaluate alternative phylogenetic and species delimitation hypotheses in a recent evolutionary radiation of grasshoppers. Mol. Ecol. 2018; 27: 1229–1244.
14. Huang JH, Zhang AB, Mao SL, Huang Y. DNA barcoding and species boundary delimitation of selected species of Chinese Acridoidea (Orthoptera: Caelifera). PLoS ONE. 2013; 8(12): e82400.
15. You QJ, Li TS, Bi DY. Description of new genera and species of Catantopidae from Guangxi (Orthoptera: Acridoidea). Entomotaxonomia. 1983; 5(2): 165–181.
16. Jiang B, Wang HJ, Storozhenko SYu, Huang JH. Notes on the grasshopper genera *Longgenacris* You and Li, 1983 and *Fruhstorferiola* Willemse, 1922 (Orthoptera: Acrididae: Melanoplinae) with proposal of a new synonym. Zootaxa. 2019; 4624 (3): 397–406.
17. Cigliano MM, Braun H, Eades DC, Otte D. Orthoptera Species File. Version 5.0/5.0. http://Orthoptera.SpeciesFile.org [Date of access: 30.01.2020].
18. Zheng ZM, Wei SZ. A new species of the genus *Longgenacris* You *et* Li from China (Orthoptera: Catantopidae). Acta Zootax. Sinica. 2003; 28(3): 482–484.
19. Yassin A, Amédégnato C, Cruaud C, Veuille M. Molecular taxonomy and species delimitation in Andean *Schistocerca* (Orthoptera:Acrididae). Mol. Phylogeneti. Evol. 2009; 53: 404–411.
20. Jesús-Bonilla VS, Riveron AZ. Species delimitation in the grasshopper genus *Taeniopoda* (Orthoptera: Romaleidae) based on molecular and morphological evidence. Genome. 2015; 58(5): 210–211.
21. Guzmán NV, Pietrokovsky SM, Cigliano MM, Confalonieri VA. Unraveling the diversification history of grasshoppers belonging to the "*Trimerotropis pallidipennis*" (Oedipodinae: Acrididae) species group: a hotspot of biodiversity in the Central Andes. PeerJ. 2017; 5: e3835.
22. Herbert PDN, Cywinska A, Ball SL, deWaard JR. Biological identifications through DNA barcodes. P. Roy. Soc. B-Biol. Sci. 2003; 270: 313–321.
23. Templeton AR, Crandall KA, Sing CF. A cladistic analysis of phenotypic associations with haplotypes inferred from restriction endonuclease mapping and DNA sequence data. III. Cladogram estimation. Genetics. 1992; 132: 619–633.
24. Pons J, Barraclough TG, Gomez-Zurita J, Cardoso A, Duran DP, Hazell S, et al. Sequencebased species delimitation for the DNA taxonomy of undescribed insects. Syst. Biol. 2006; 55: 595–609.
25. Li HC, Xia KL. Fauna Sinica, Insecta, vol. 43, Orthoptera, Acridoidea, Catantopidae. Beijing, China: Science Press; 2006. Chinese.
26. Tian YF, Huang G, Zheng ZM, Wei ZM. A simple method for isolation of insect total DNA. J. Shaanxi Norm. Univ. (Nat. Sci. Ed.). 1999; 27: 82–84.
27. Ji YJ, Zhang DX, He LJ. Evolutionary conservation and versatility of a new set of primers for

amplifying the ribosomal internal transcribed spacer regions in insects and other invertebrates. Mol. Ecol. Notes. 2003; 3: 581–585.
28. White TJ, Bruns T, Lee S, Taylor JW. Amplification and direct sequencing of fungal ribosomal RNA genes for phylogenetics. In: Innis MA, Gelfand DH, Sninsky JJ, White TJ, editors. PCR protocols, a guide to methods and applications. London: Academic Press, Inc.; 1990. p. 315-322.
29. Staden R, Beal KF, Bonfield JK. The Staden Package, 1998. Comput. Methods Mol. Biol. 2000; 132: 115–130.
30. Thompson JD, Gibson TJ, Plewniak F, Jeanmougin F, Higgins DG. The CLUSTAL_X window interface: Flexible strategies for multiple sequence alignment aided by quality analysis tools. Nucleic Acids Res. 1997; 25: 4876–4882.
31. Trewick SA. DNA Barcoding is not enough: mismatch of taxonomy and genealogy in New Zealand grasshoppers (Orthoptera: Acrididae). Cladistics. 2008; 24: 240–254.
32. Song H, Moulton MJ, Whiting MF. Rampant nuclear insertion of mtDNA across diverse lineages within Orthoptera (Insecta). PLoS ONE. 2014; 9: e110508.
33. Moulton MJ, Song H, Whiting MF. Assessing the effects of primer specificity on eliminating numt coamplification in DNA barcoding: a case study from Orthoptera (Arthropoda: Insecta). Mol. Ecol. Resour. 2010; 10: 615–627.
34. Vaidya G, Lohman D J, Meier R. SequenceMatrix: concatenation software for the fast assembly of multi-gene datasets with character set and codon information. Cladistics. 2011; 27(2): 171–180.
35. Kimura M. A simple method for estimating evolutionary rate of base substitutions through comparative studies of nucleotide sequences. J. Mol. Evol. 1980; 16: 111–120.
36. Nei M, Kumar S. Molecular evolution and phylogenetics. London: Oxford University Press; 2000.
37. Kumar S, Stecher G, Tamura K. MEGA7: Molecular Evolutionary Genetics Analysis version 7.0 for bigger datasets. Mol. Biol. Evol. 2016; 33: 1870–1874.
38. Nguyen L-T, Schmidt HA, Haeseler A, Minh BQ. IQ-TREE: A fast and effective stochastic algorithm for estimating maximum-likelihood phylogenies. Mol. Biol. Evol. 2014; 32(1): 268–274.
39. Kalyaanamoorthy S, Minh BQ, Wong TKF, Haeseler A, Jermiin LS. ModelFinder: Fast model selection for accurate phylogenetic estimates. Nat. Methods. 2017; 14: 587–589.
40. Hoang DT, Chernomor O, Haeseler A, Minh BQ, Vinh LS. UFBoot2: Improving the ultrafast bootstrap approximation. Mol. Biol. Evol. 2018; 35: 518–522.
41. Trifinopoulos J, Nguyen L-T, Haeseler A, Minh BQ. W-IQ-TREE: a fast online phylogenetic tool for maximum likelihood analysis. Nucleic Acids Res. 2016; 44: W232–235.
42. Saitou N, Nei M. The neighbour-joining method: a new method for reconstructing evolutionary trees. Mol. Biol. Evol. 1987; 4: 406–425.
43. Kumar S, Gadagkar SR. Efficiency of the neighbor-joining method in reconstructing deep and shallow evolutionary relationships in large phylogenies. J. Mol. Evol. 2000; 51: 544–553.
44. Posada D, Crandall KA. Intraspecific genegenealogies: trees grafting into networks. Trends Ecol. Evol. 2001; 16: 37–45.
45. Templeton AR. Using phylogeographic analyses of gene trees to test species status and processes. Mol. Ecol. 2001; 10: 779–791.

46. Hart MW, Sunday J. Things fall apart: Biological species form unconnected parsimony networks. Biol. Letters. 2007; 3: 509–512.
47. Clement M, Posada D, Crandall KA. TCS: a computer program to estimate gene genealogies. Mol. Ecol. 2000; 9: 1657–1659.
48. Drummond A J, Suchard MA, Xie D, Rambaut A. Bayesian phylogenetics with BEAUti and the BEAST 1.7. Mol. Biol. Evol. 2012; 29: 1969–1973.
49. Paz A, Crawford AJ. Molecular-based rapid inventories of sympatric diversity: A comparison of DNA barcode clustering methods applied to geography-based vs clade-based sampling of amphibians. J. Biosciences. 2012; 37: 887–896.
50. Pentinsaari M, Vos R, Mutanen M. Algorithmic single-locus species delimitation: Effects of sampling effort, variation and nonmonophyly in four methods and 1870 species of beetles. Mol. Ecol. Resour. 2016; 17: 393–404.
51. Esselstyn JA, Evans BJ, Sedlock JL, Khan FAA, Heaney LR. Single-locus species delimitation: A test of the mixed Yule-coalescent model, with an empirical application to Philippine round-leaf bats. P. Roy. Soc. B-Biol. Sci. 2012; 279: 3678–3686.
52. Tänzler R, Sagata K, Surbakti S, Balke M, Riedel A. DNA Barcoding for community ecology - how to tackle a hyperdiverse, mostly undescribed melanesian fauna. PlosONE. 2012; 7: e28832.
53. Fujisawa T, Barraclouch T. Delimiting species using single-locus data and the generalized mixed Yule coalescent approach: a revised method and evaluation on simulated data sets. Syst. Biol. 2013; 62(5): 707–724.
54. Talavera G, Dinca V, Vila R. Factors affecting species delimitations with the GMYC model: insights from a butterfly survey. Methods Ecol. Evol. 2013; 4: 1101–1110.
55. Dasmahapatra KK, Ellas M, Hill RI, Hoffmans JI, Mallet J. Mitochondrial DNA barcoding detects some species that are real, and some that are not. Mol. Ecol. Resour. 2010; 10: 264–273.
56. Thielsch A, Knell A, Mohammadyari A, Petrusek A, Schwenk K. Divergent clades or cryptic species? Mitonuclear discordance in a Daphnia species complex. BMC Evol. Biol. 2017; 17, 227.
57. Yang LH, Konga H, Huang J P, Kang M. Different species or genetically divergent populations? Integrative species delimitation of the *Primulina hochiensis* complex from isolated karst habitats. Mol. Phylogenet. Evol 2019; 132, 219–231.
58. Dayrat B. Towards integrative taxonomy. Biol. J. Linn. Soc. 2005; 85, 407–415.

Captions:

Fig 1 Shape of male cerci in *Fruhstoferiola* spp. A. *F. viridifemorata*. B. *F. tonkinensis*. C. *F. huangshanensis*.

Fig 2 Variation of teeth in posterior margins of female subgenital plates of *Fruhstorferiola* spp. A-B, J. *F. viridifemorata*. C-D, I. *F. omei*. E-F. *F. kulinga*. G-H. *F. huayinensis*. A, C, E, G. The condition with median tooth distinctly longer than submedian and lateral teeth.B, D, F, H. The

condition with median tooth nearly as long as submedian and lateral teeth. I. The condition with median tooth slightly shorter than submedian and lateral teeth. J. The condition with submedian teeth absent.

Fig 3 Sample and type localities of *Fruhstorferiola* spp.

Fig 4 Phylogeny deduced in maximum likelihood framework from alignment of COI gene and concatenated alignment of COI gene, ITS1 and ITS2 sequences. A–C. Cladogram deduced from COI gene. D–F. Cladogram deduced from concatenated alignment of COI gene, ITS1 and ITS2 squences. A, D. Full trees with subclade of *L. rufiantennus*+*F. tonkinensis* group and *F. viridifemorata* group collapsed. B, E. Subclades of *L. rufiantennus*+*F. tonkinensis* group. C, F. Subclade of *F. viridifemorata* group.

Fig 5 NJ tree and haplotype networks of *F. tonkinensis*+*L. rufiantennus* group. A. Subclade of NJ tree for *F. tonkinensis*+*L. rufiantennus* group reconstructed from COI gene. B. Haplotype network reconstructed from COI gene, C. Haplotype network reconstructed from ITS1 sequence. D. Haplotype network reconstructed from ITS2 sequence (including *F. viridifemorata* and *L. maculacarina*).

Fig 6 Species delimitation according to the generalized mixed Yule coalescent (GMYC) single-threshold model using COI data set. A. Lineage-through-time plot based on the ultrametric tree obtained from COI sequences. The sharp increase in branching rate, corresponding to the transition from interspecific to intraspecific branching events, is indicated by a red vertical line. The *x*- axes (both in panels a and b) show substitutions per nucleotide site. B. Likelihood function produced by GMYC to estimate the peak of transition between cladogenesis (interspecific diversification) and allele intraspecific coalescence along the branches. C. *F. tonkinensis*+*L. rufiantennus* subclade of the ultrametric tree. D. *F. viridifemorata* group subclade of the ultrametric tree.

Fig 7 NJ tree and haplotype networks of *F. viridifemorata* group. A. Subclade of NJ tree for *F. viridifemorata* group reconstructed from COI gene. B. Haplotype network reconstructed from COI gene, C. Haplotype network reconstructed from ITS1 sequence. D. Haplotype network reconstructed from ITS2 sequence.

Figure 1

Click here to download Figure Fig1.tif

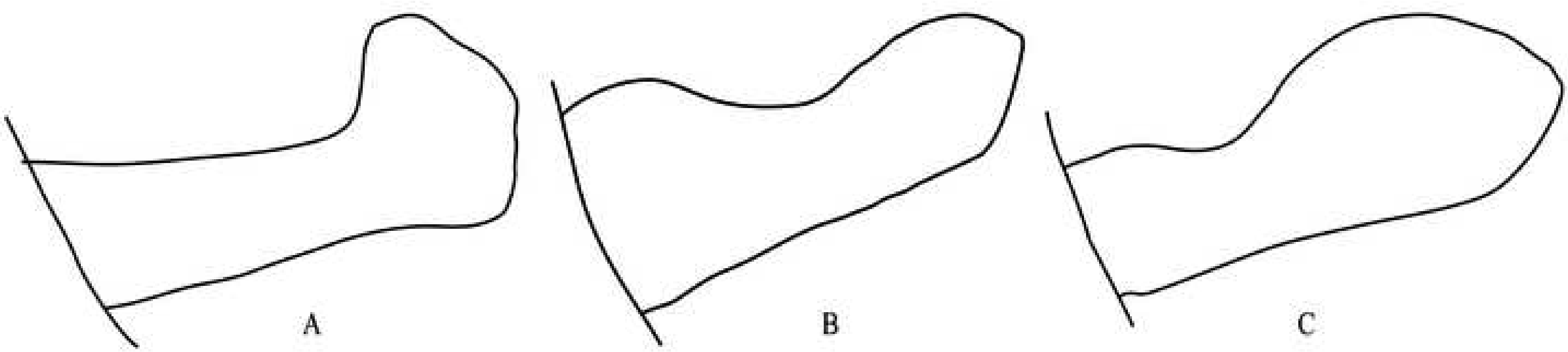

Click here to download Figure Fig2.tif

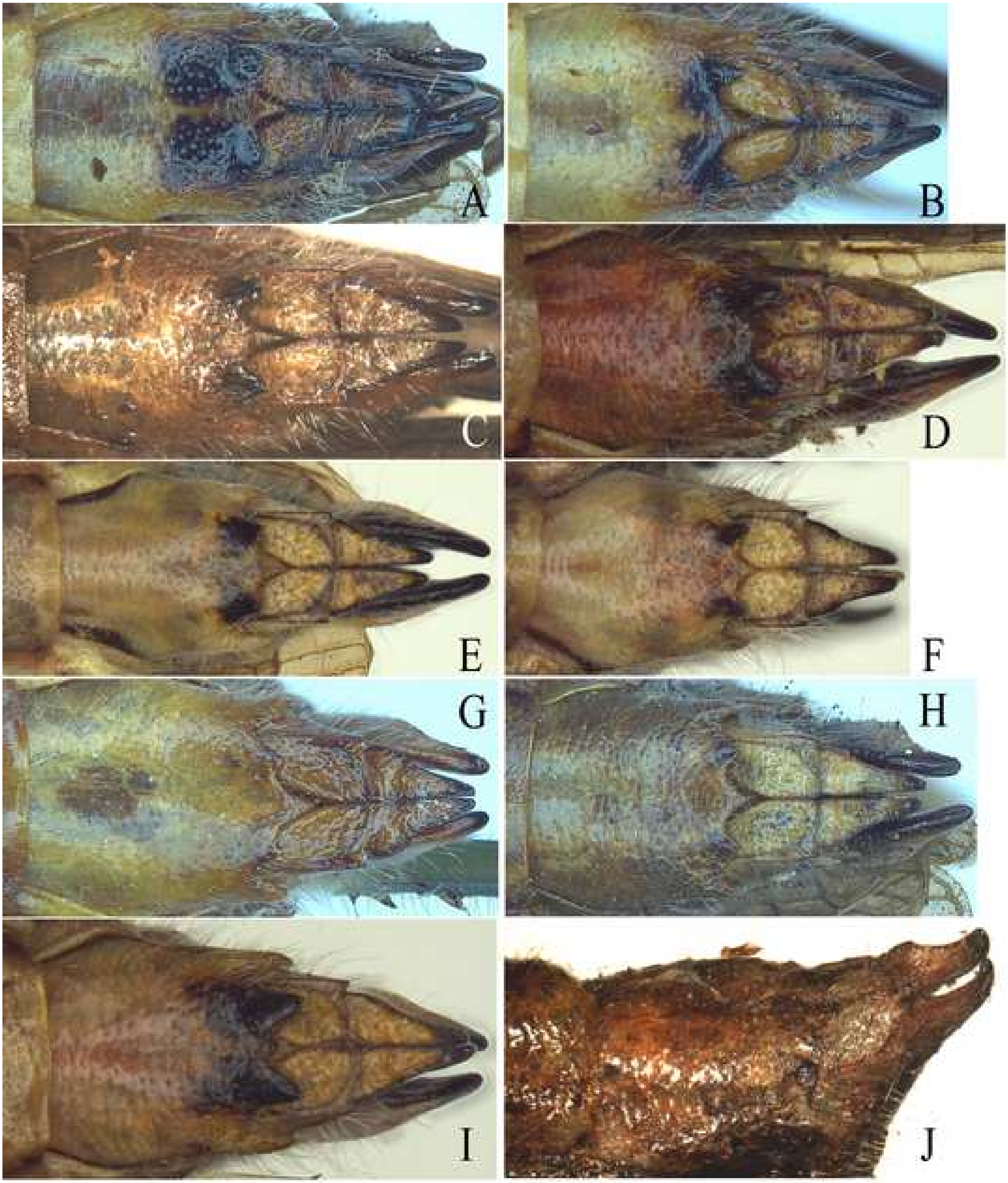

Figure 3

Click here to download Figure Fig3.tif

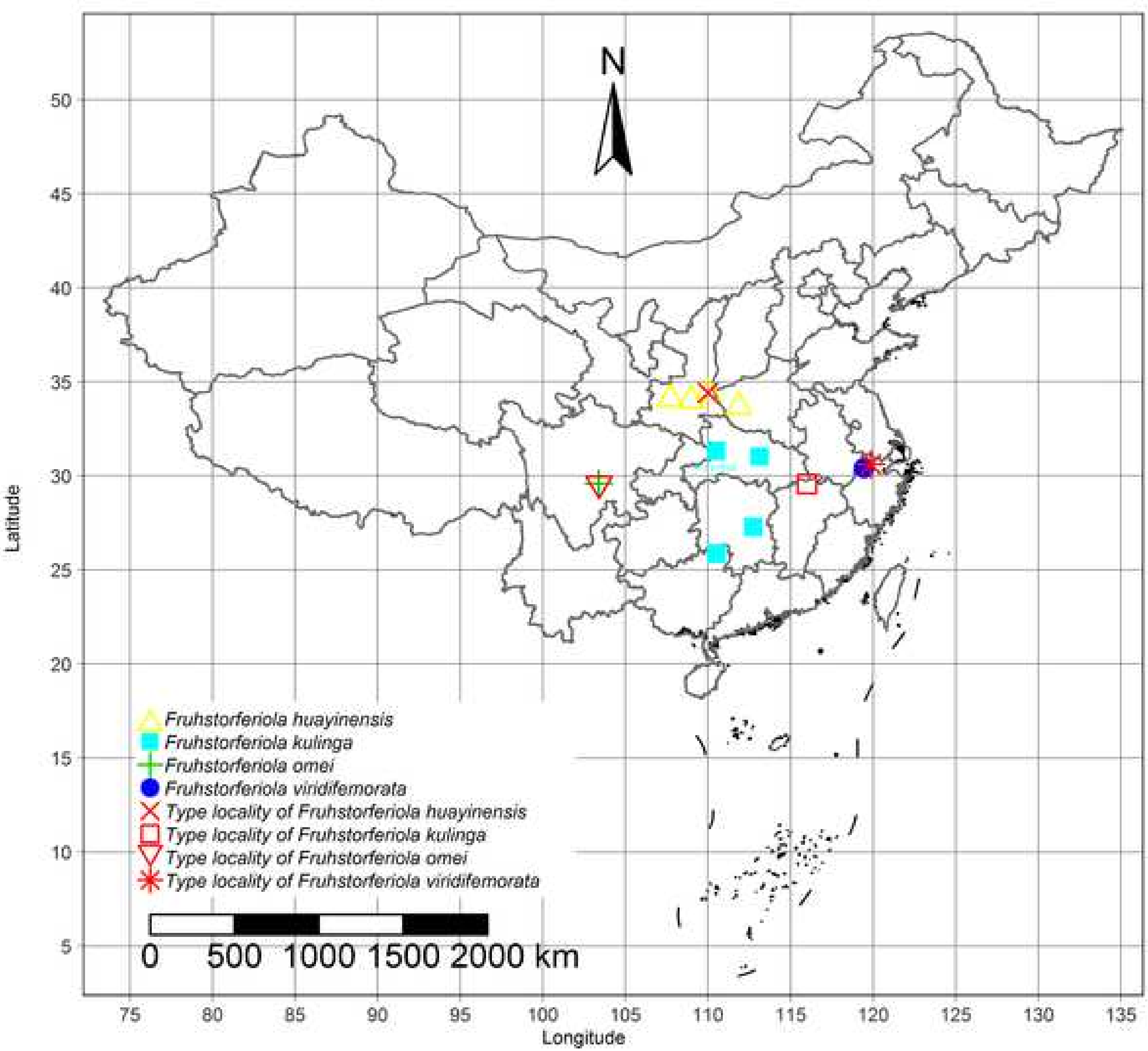

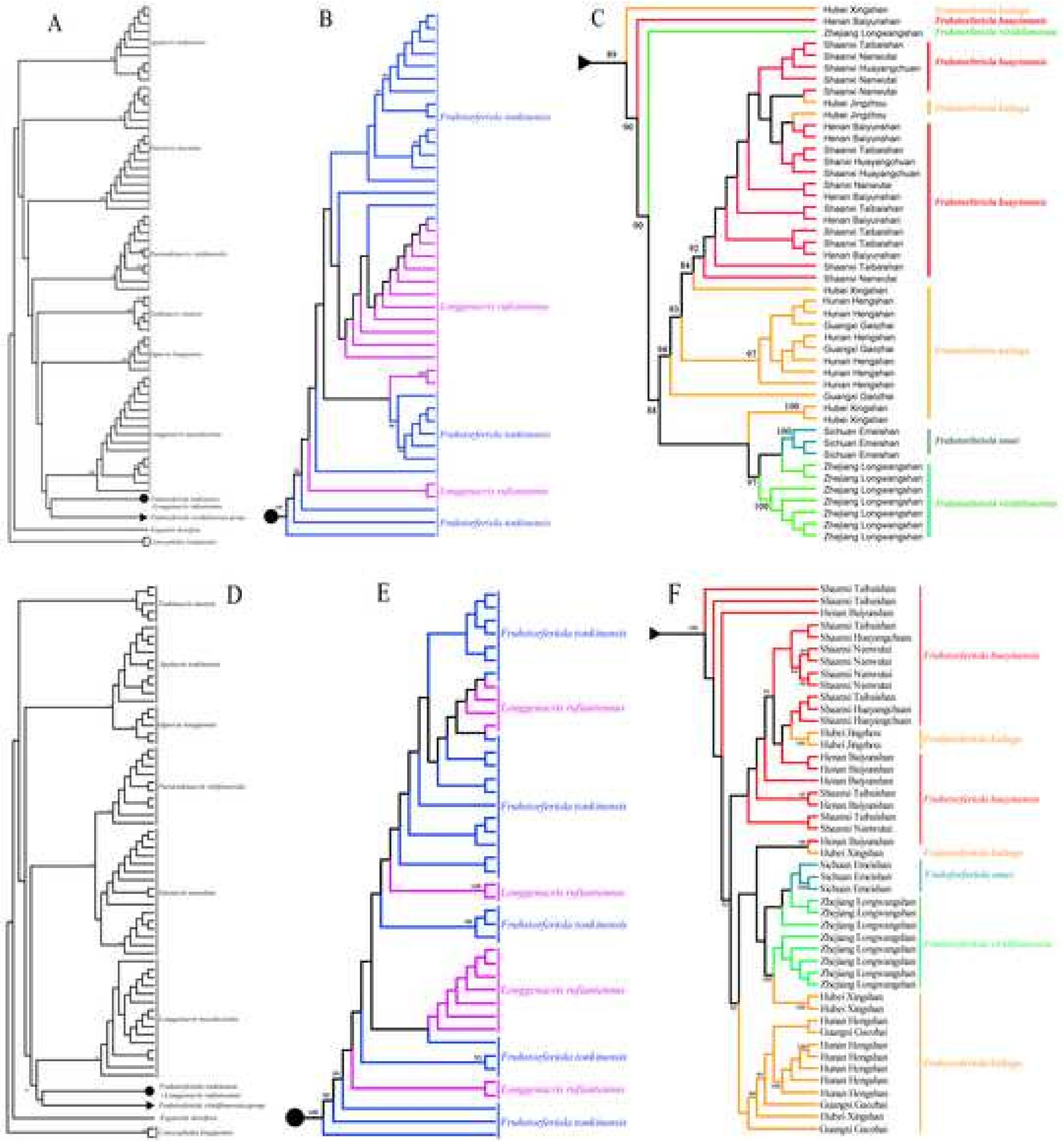
A
B
C
D
E
F

Figure 5

Click here to download Figure Fig5.tif

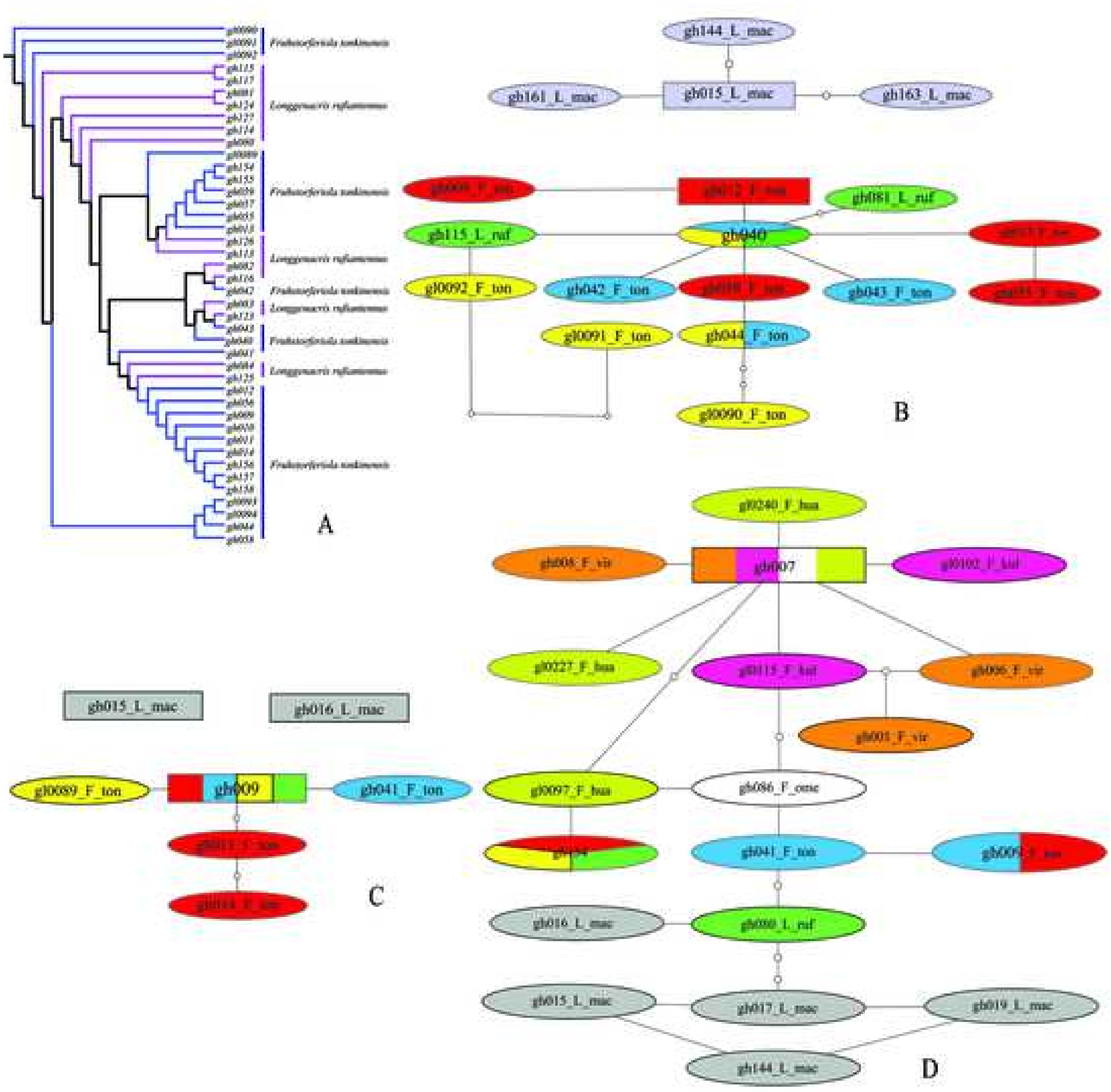

Figure 6

Click here to download Figure Fig6.tif

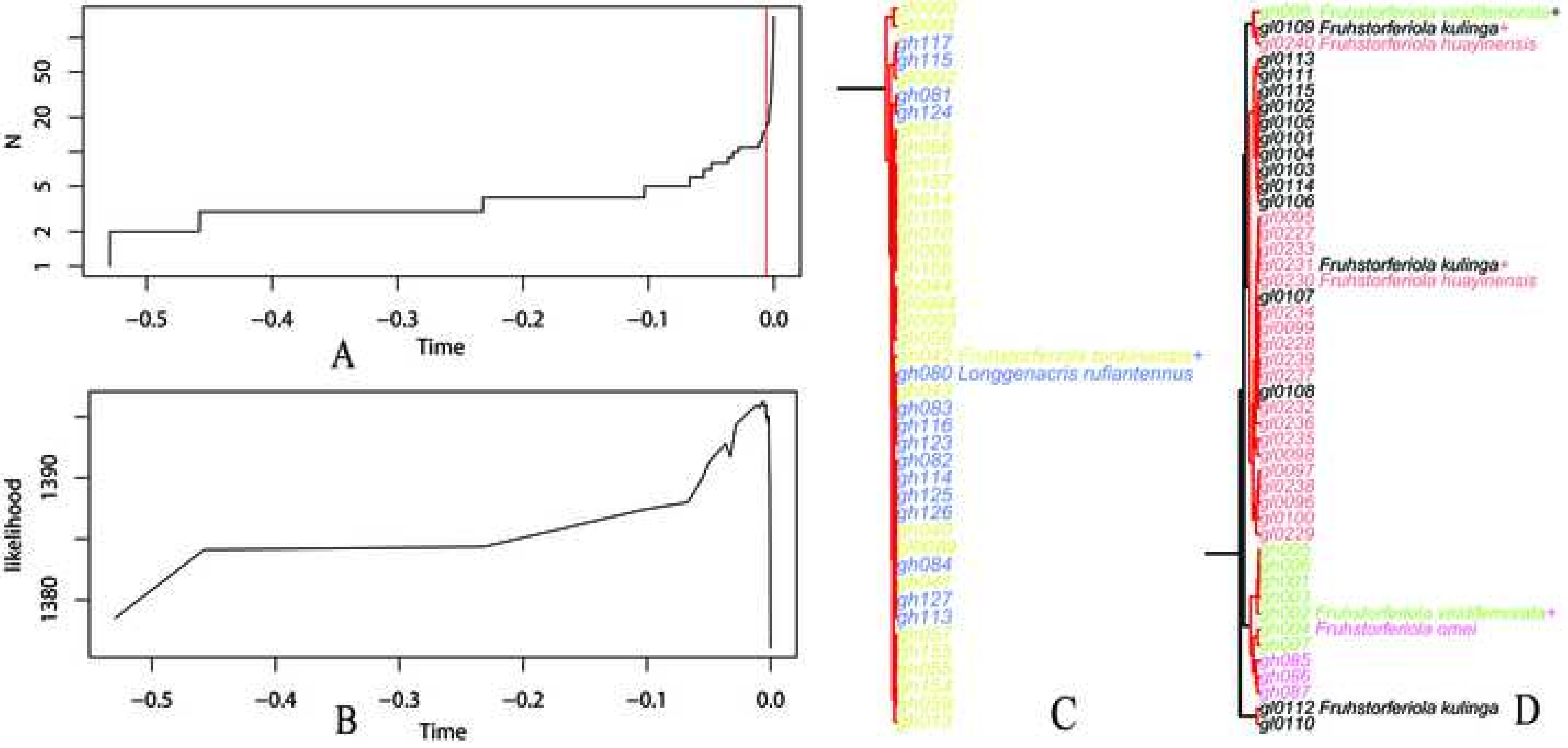

 Click here to download Figure Fig7.tif

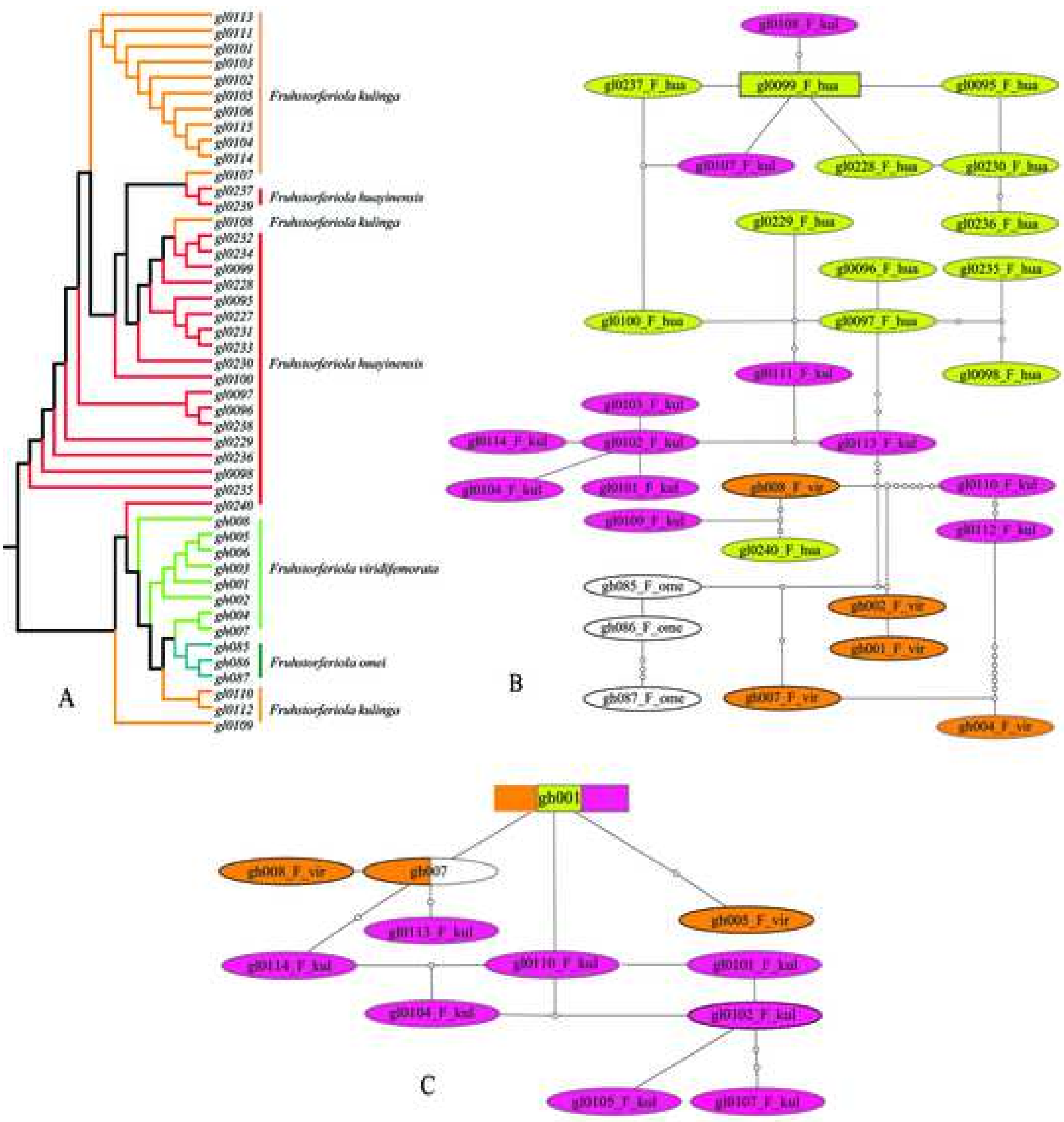

S1 Table

Click here to access/download
Supporting Information
S1 Table-Materials involved in this study.docx

S2 Table

Click here to access/download
Supporting Information
S2 Table-Mapping table.docx

S3 Table

Click here to access/download
Supporting Information
S3 Table-Intraspecific variations.docx

S4 Table

Click here to access/download

Supporting Information

S4 Table-Distances between species-COI.docx

S5 Table

Click here to access/download
Supporting Information
S5 Table-distances between species-ITS1.docx

Click here to access/download
Supporting Information
S6 Table-distances between species-ITS2.docx

S7 Table

Click here to access/download
Supporting Information
S7 Table-Haplotyptes of COI.docx

S8 Table

Click here to access/download
Supporting Information
S8 Table-Haplotyptes of ITS1.docx

S9 Table

Click here to access/download
Supporting Information
S9 Table-Haplotyptes of ITS2.docx

S10 Table

Click here to access/download

Supporting Information

S10 Table-Species delimitation by GMYC.docx

S1 Fig

Click here to access/download
Supporting Information
S1 Fig-Phylogeny from ITS1.docx

S2 Fig

Click here to access/download
Supporting Information
S2 Fig-Phylogeny from ITS2.docx

S3 Fig

Click here to access/download
Supporting Information
S3 Fig-NJ trees.docx

S4 Fig

Click here to access/download
Supporting Information
S4 Fig-Species delimitation by GMYC.docx
