# Supplementary material for: Phylogeny and species delimitation of the genus Longgenacris and Fruhstorferiola viridifemorata species group (Orthoptera: Acrididae: Melanoplinae) based on molecular evidence
Source: PLoS One. 2020 Aug 26;15(8):e0237882. doi: 10.1371/journal.pone.0237882 (PMC7449498; doi:10.1371/journal.pone.0237882)
Supplement: Attachment — Submitted filename: Response to reviewers.docx [file pone.0237882.s016.docx]

Dear Editor,

Below is a rebuttal letter that responds to each point raised by the academic editor and reviewer(s).

1. Gene names must be set into italics. We have set all gene names in the manuscript into italics.

2. The letter "l" in the volume unit "μl" in the section "DNA extraction, PCR amplification and sequencing" have been revised from lower case "l" into upper case "L".

3. References for IQ-TREE and W-IQ-TREE. We have cited the reference for IQ-TREE，but not in the place the reviewer marked, and now we have changed citation of the reference to the place the reviewer marked.

4. Adds Bayesian inference analysis. We have reconstructed BI tree using for different datasets, but the results is similar to ML tree and have no significant difference. Therefore, we think there is no need to add the figures of BI trees to the manuscript. Below are four selected figures of the BI trees.

**BI tree reconstructed from COI dataset.** Left: full tree with the subclade of *Fruhstorferiola spp* collapsed. right: the subclade of *Fruhstorferiola spp*.

**BI tree reconstructed from combined dataset.** Left: full tree with the subclade of *Fruhstorferiola spp* collapsed. right: the subclade of *Fruhstorferiola spp*.

5. Other minor issues the reviewer pointed out. We have carefully revised all issues the reviewer pointed out.

(1) line 36. The keyword "Longgenacris rufiantennus" has been deleted.

(2) line 104. "cytochrome c oxidase subunit I (COI, cox1)" has been replaced with "COI " onlyy.

(3) line 122 . "100% ethanol" has been revised into "anhydrous ethanol".

(4) line 140-141. "PCR products were sent to the biological company", Which company？the full name of the biological company has bee previded in the text.

(5) line 183 "TSC1.21" has been revised into "TCS1.21".

6. Using Tetrigidae and Tettigoniidae as outgroups is not a right choice. Outgroup(s) is only a reference group to improve further the acuracy of the topology inferred. So, a group of any taxa should be appropriate outgroup only if it does not decrease the quality of the tree topology. In our study, selecting Tetrigidae and Tettigoniidae as outgroups is feasible because no such problem occurred in our study.

7. Normally, as for the raltionship between species and above, NJ tree is not optimal selection. As we mentioned in the section "Intraspecific variation, interspecific divergence and phylogeny reconstruction", the main object of NJ tree is to provide a profile for the setup of taxa and groups for calculating genetic distances, and to provide a graphic representation of the patterning of divergence between species because of its strong track record in the analysis of large species assemblages. We did not aim to resolve the relationship above species level using NJ tree. But it will be useful when synonymies may exist among a group of species-level taxa. In our manuscript, the NJ trees were mainly used to clarify the relationships among extremely closely related species when performing species delimitation but not among distantly related taxa.

8. On the page 16, line 403, “ITS region had a much lower evolution rate than COI gene in our datasets”, I’m very interested in it. Can the author explain this? At the moment, we cannot be able to explain such a special case, but we will follow with interest in the future.

9. All figures are not clear. Low photo resolution. This is not the case. All figures we uploaded have enough resolution. the figures the reviewer referred with low resolution are the version compressed when the pdf draft was created by the system.

10. English speakers are requested to polish this draft.
